# Genetic diversity assessed by genotyping by sequencing (GBS) and for phenological traits in blueberry cultivars

**DOI:** 10.1371/journal.pone.0206361

**Published:** 2018-10-23

**Authors:** Ana Campa, Juan José Ferreira

**Affiliations:** Plant Genetics, Area of Horticultural and Forest Crops, SERIDA, Asturias, Spain; Università Politecnica delle Marche, ITALY

## Abstract

Blueberry is a small fruit crop which includes a complex group of different *Vaccinium* species of various ploidy levels. Commercial blueberries have been grown in Europe most recently, so there is not much information available about their adaptation into new regions. In this work we investigated adaptation to the environmental conditions of northern Spain, in terms of flowering and ripening seasons, of a set of 70 blueberry cultivars including several of the most important cultivated American species (*V*. *corymbosum*, *V*. *virgatum*, *V*. *macrocarpon* and *V*. *uliginosum*) in order to identify which types are best-suited in this geographical area of Europe. Most materials showed high chilling requirements for flowering under local conditions, while materials with low-chilling requirements showed problems in the maturation process of the flowers. Most cultivars were early or mid-season while a relative lack of late-season cultivars was observed. GBS was used for the analysis of genetic diversity in this sample of 70 cultivars. A total of 5255 SNP markers were obtained and a cluster analysis revealed three main groups associated with the ploidy level of the species. A Principal Component Analysis revealed a grouping of the *V*. *corymbosum* cultivars according to their chilling requirements. A total of 29 SNPs were identified as being highly informative for diversity analysis and potentially useful for cultivar identification and for breeding purposes. The results obtained from this research should contribute to the expansion of this crop, as well as providing data about genetic diversity useful for the preservation of genetic resources or for future breeding programs.

## Introduction

Blueberries (*Vaccinium* section *Cyanococcus*) are perennial shrubs and small fruit crops, production of which has risen quickly around the world [1] associated with an increase in consumer demand due to their recognized health benefits [2,3,4]. The fruit is a small, pulpy, and indigo-colored berry, 10–16 mm in diameter, and distributed in more or less dense clusters. Blueberries have a high concentration of phenolic compounds and anthocyanins [5], which are known for their high antioxidant capacity that neutralizes free radicals and can potentially provide protection against carcinogenicity, cardiovascular and neurodegenerative changes associated with aging [6,7,8]. In certain blueberry cultivars (cvs) high concentrations of iridoid glycosides, a large group of secondary metabolites with potential human health benefits, have also been identified [9].

Blueberries include more than 400 species of the genus *Vaccinium*, family *Ericaceae* [10]. The main cultivated species around the world are of American origin [11,12]: *V*. *corymbosum* L., known as highbush blueberries; *V*. *virgatum* Aiton (syn. *V*. *ashei* Reade), or rabbiteye; *V*. *angustifolium* Aiton, known as lowbush blueberries; *V*. *macrocarpum* Aiton known as American cranberry. Hybrid cvs obtained from interspecific crosses are also grown, such as the so called half-highs that are hybrids between highbush and lowbush blueberry species [11]. Blueberries are usually classified into three groups depending on their chilling requirements for the initiation of flowering, measured as accumulated hours of temperature <7ºC [11]: (i) cvs with high chilling requirements (more than 800 h chilling), typically grown in cold winter regions. Highbush cvs with these requirements are named northern highbush. Half-high hybrids and lowbush blueberries generally have high chilling requirements; (ii) cvs with moderate-chilling requirements (between 500–800 h) including most rabbiteyes, grown in regions with temperate winters; and (iii) cvs with low-chilling requirements (less than 500 h) grown in regions with mild winters. Highbush varieties with low-chilling requirements are named southern highbush blueberries, and are interspecific hybrids derived from crosses between highbush and species native to the southeastern United States with lower chilling requirements, such as *V*. *darrowii* Camp. [13,14,15].

Blueberry species occur in nature at different ploidy levels (2n = 2x = 24), which increases the complexity of this genus [16,17]. The species *V*. *corymbosum*, *V*. *angustifolium* and *V*. *uliginosum* are tetraploid, *V*. *virgatum* is hexaploid, and *V*. *macrocarpum* is diploid. The ploidy is autopolyploidy, with more than two genetically similar (homologous) genomes derived from chromosome doubling of the same genome [18,19]. All blueberry species are cross-pollinated, although certain varieties are considered to be self-fertile [20]. Interspecific hybridization has played a major role in blueberry breeding, incorporating novel traits from wild germplasm. Blueberry improvement started in 1909 in EEUU, in parallel to domestication, through selection of wild populations and cross-pollinations [21,22,23]. First breeding efforts were focused on the development of cvs with broader climatic and soil adaptation, tolerance of mechanical handling, and improved fruit quality [24]. Currently, there are many varieties available, e. g. 117 *V*. *corymbosum* and 12 *V*. *virgatum* cvs have been recorded in the European Community Plant Variety Office (https://cpvoextranet.cpvo.europa.eu). However, this industry has grown so rapidly that there is little information concerning the agronomic value of the cvs in different geographical areas, while the low phenotypic polymorphism between cvs with respect to plant, leaf or fruit traits renders cv identification difficult. Differentiation of blueberry cvs requires detailed evaluation in the field over several years.

Variation supplied by molecular markers can help assessment of genetic diversity and cv identification, or to achieve genetic analysis or marker assisted selection. But the use of molecular markers in blueberry research was limited until the recent advent of next-generation sequencing (NGS) technologies, due to the complexity of this group [25,26,27]. Most of the genomic data obtained to date for blueberry have been summarized and are available on different websites [28]. A genome-sequencing project of a diploid *V*. *macrocarpum* cv is underway, assembled as scaffolds [29]. Genotyping-by-sequencing (GBS) has emerged as a genomic approach for exploring plant genetic diversity of complex species lacking extensive genomic resources [30]. GBS has been applied to massive genotyping and genetic diversity analyses in polyploid crops such as potato [31], coffee [32] and wheat [33]. To date, GBS in *Vaccinium* spp has been applied to the development of two high-density linkage maps, one in a diploid *V*. *macrocarpum* population [34] and the other in a tetraploid *V*. *corymbosum* population [27].

One of the most important aspects for a successful expansion of the blueberry crop is the selection of cvs, which basically depends on knowledge about their adaptation to local conditions. Commercial blueberries have been grown in Europe since only the 1930s, so there is not much information available about their adaptation into new geographical regions. One of the aims of this work was to investigate the adaptation to the environmental conditions of northern Spain, in terms of flowering and ripening seasons, of a set of 70 commercial blueberry cvs of North American origin in order to identify which types are best suited to conditions in northern Spain or to similar latitudes. For breeders, it is important to know the genetic diversity among cvs and accessions, as a low level of variation is expected in progeny derived from crosses between closely related parents. Other aim of this study was to investigate the genetic diversity included in the set of 70 cvs using the single nucleotide polymorphism (SNP) variation supplied by GBS. This information will be useful for growers, for optimize breeding efforts and for the utilization and preservation of blueberry genetic diversity.

## Results

### Phenological traits

**Fig 1:**
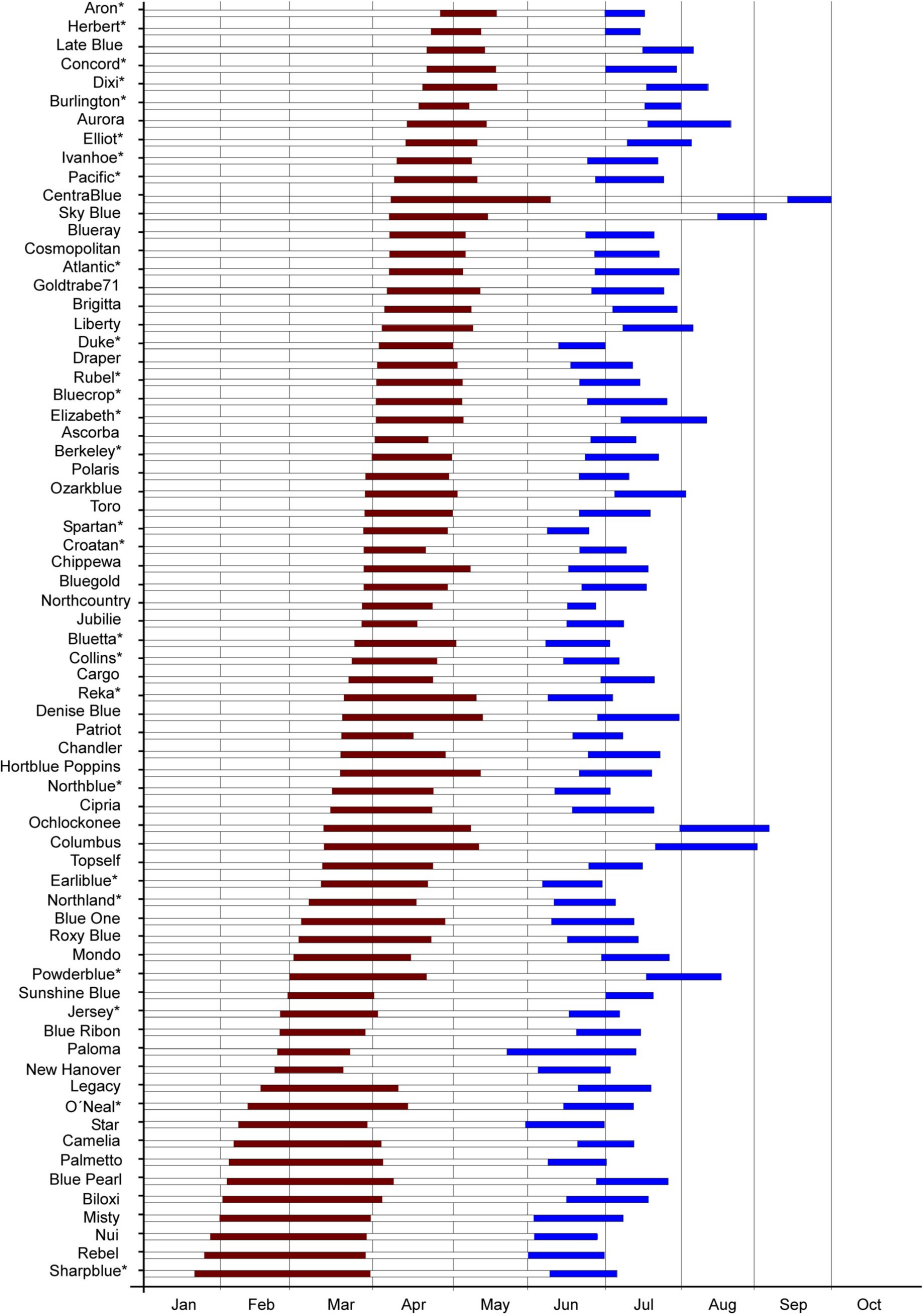
Bar graph showing flowering and fruit season. Flowering and fruit season per cultivar estimated in local growing conditions for years 2012 to 2017. Red bars represent flowering season and blue bars represents harvest season. Varieties are sorted by beginning of flowering.*. varieties included in the NCGR-USDA *Vaccinium* Core Collection.

Fig 1 presents the flowering and harvesting seasons estimated for each cv based on data scored over six years (see S1 Table). The first cv to start flowering was Sharpblue (20 d from 1January) and the last one was Aron (118 d from 1 January). Based on this phenological trait, the 70 cvs were classified as having high, moderate or low chilling requirements (Table 1). The species *V*. *uliginosum* showed a high chilling requirement. The rabbiteye and the half-high hybrid groups showed variation for this trait, and included cvs exhibiting high, moderate or low chilling requirement. Within the *V*. *corymbosum* group, most cvs had high chilling requirement, though 16 exhibited low chilling requirement, and only six were classified as mid chilling. There were also differences between cvs with respect to the duration of flowering season (Fig 1, S1 Table), ranging from 66 d of Sharpblue and Blue Pearl to 19 d for Herbert, with a mean flowering season duration of 39 d.

**Table 1:** List of blueberry cultivars analyzed in this work.

Cultivar^a^	Species^b^	Pedigree^b^	Chill requirements	Harvest season
Pilgrim*	Vm	Prolific x McFarlin	-	-
Aron*	Vu	-	high	mid
Centrablue	Vv	Centurion x Rahi	high	extra-late
Columbus	Vv	-	mid	late
Ochlockonee	Vv	Tifblue (Ethel x Clara) x Menditoo (Myers x Black Giant)	mid	late
Powderblue*	Vv	Tifblue (Ethel x Clara) x Menditoo (Myers x Black Giant)	low	mid
Sky Blue	Vv	Centurion x Rahi	high	late
Chippewa	HH	B18A (G65 x Ashworth) x US3 (Dixi x Michigan lowbush1)	high	mid
Northblue*	HH	B10 (G65 x Ashworth) x US3 (Dixi x Michigan lowbush1)	mid	early
Northcountry	HH	B6 (G65 x Asworth) x R2P4 (Vc *x V*. *angustiflolium*)	high	mid
Northland*	HH	Berkeley x 19-H (lowbush x Pioneer)	mid	early
Polaris	HH	G65 x Asworth	high	mid
Sunshine Blue	HH	-	low	mid
Ascorba	Vc	-	high	mid
Atlantic*	Vc	Jersey x Pioneer	high	mid
Aurora	Vc	Brigitta Blue x Elliot	high	mid
Berkeley*	Vc	Stanley x GS149 (Jersey x Pioneer)	high	mid
Biloxi	Vc	Sharpblue x US329	low	mid
Blue One	Vc	-	mid	early
Blue Pearl	Vc	-	low	mid
Blue Ribon	Vc	G344 x Toro	low	mid
Bluecrop*	Vc	GM37 (Jersey x Pioneer) x CU5 (Stanley x June)	high	mid
Bluegold	Vc	Bluehaven x Me-US5 (Ashworth x Bluecrop)	high	mid
Blueray	Vc	(Jersey x Pioneer) x (Stanley x June)	high	mid
Bluetta*	Vc	(North Sedgwick x Coville) x Earlyblue	high	early
Brigitta	Vc	-	high	mid
Burlington*	Vc	Rubel x Pioneer	high	mid
Camellia	Vc	MS-122 (G144 x US121) x MS-6 (G107 x Sharpblue)	low	mid
Cargo	Vc	-	high	mid
Chandler	Vc	Darroe x M-23	high	mid
Cipria	Vc	-	mid	mid
Collins*	Vc	Stanley x Weymouth	high	early
Concord*	Vc	Brooks x Rubel	high	mid
Cosmopolitan	Vc	-	high	mid
Croatan*	Vc	Weymouth x F-6 (Stanley x Crabbe4)	high	mid
Denise Blue	Vc	-	high	mid
Dixi*	Vc	(Jersey x Pioneer) x Stanley	high	mid
Draper	Vc	Duke x (290–2 x MSU652)	high	mid
Duke*	Vc	(Ivanhoe x Earliblue) x 192–8 (E-30 x E-11)	high	early
Earliblue*	Vc	Stanley x Weymouth	mid	early
Elizabeth*	Vc	(Katharine x Jersey) x Scammel	high	mid
Elliott*	Vc	Burlington x (Dixi x (Jersey x Pioneer))	high	mid
Goldtraube 71	Vc	-	high	mid
Herbert*	Vc	Stanley x GS149 (Jersey x Pioneer)	high	mid
Hortblue Poppins	Vc	Nui x 1386	high	mid
Ivanhoe*	Vc	(Rancocas x Carter) x Stanley	high	mid
Jersey*	Vc	Rubel x Grover	low	mid
Jubilie	Vc	-	high	early
Late Blue	Vc	Herbert x Coville	high	mid
Legacy	Vc	Elizabeth x US 75 (*V*. *darrowi* Fla4B x Bluecrop)	low	mid
Liberty	Vc	Brigitta Blue x Elliot	high	mid
Misty	Vc	FL67-1 x Avonblue	low	early
Mondo	Vc	-	low	mid
New Hanover	Vc	NC 1522 × 'O’Neal'	low	early
Nui	Vc	(Asworth x Earliblue) x Bluecrop	low	early
O´Neal*	Vc	Wolcott x Fla4-15 (hybrid)	low	early
Ozarkblue	Vc	G-144 x 4–76	high	mid
Pacific*	Vc	Pioneer x Grover	high	mid
Palmetto	Vc	US-158 (hybrid *V*. *darrowi* x Vc) x TH-157	low	early
Paloma	Vc	-	low	early
Patriot	Vc	(Dixi x Michigan LB-1) x Earliblue	High	mid
Rebel	Vc	FL 92–84 (Primadonna) female parent	low	early
Reka*	Vc	E118 (Asworth x Earliblue) x Bluecrop	high	early
Roxy Blue	Vc	-	mid	mid
Rubel*	Vc	Wild selection of V. corymbosum	high	mid
Sharpblue*	Vc	Fla 61–5 x Fla 62–4	low	early
Spartan*	Vc	Earliblue x US 11–93	high	early
Star	Vc	FL80-31 x O´Neal	low	early
Topself	Vc	-	mid	mid
Toro	Vc	Earliblue x Ivanhoe	high	mid

Species and pedigree data of each cultivar is indicated when available. Classification of cultivars according to flower beginning (low, mid and high chilling requirements) and harvest beginning (early, mid, late and extra-late) is indicated.

^a^ Asterisk in superscript indicate accessions included in the NCGR-USDA *Vaccinium* Core Collection

^b^ Vm, *Vaccinium macrocarpon*; Vc, *Vaccinium corymbosum*; Vv: *Vaccinium virgatum;* Vu, *Vaccinium uliginosum*; HH: half-high hybrid *V*. *corymbosum* x *V*. *angustifolium*.

With respect to harvesting date traits, the first cv to be harvested was Paloma, 143 d from 1 January, and the last one was Centrablue, after 257 d. Based on this trait, 48 cvs were classified as mid-season, 19 as early-season, but only three as late-season all of them being of the rabbiteye type (Columbus, Ochlockonee and Sky Blue). The rabbiteye Centrablue was the only extra-late cv identified. There were differences between cvs with respect to the duration of the harvesting season (Fig 1, S1 Table), from 14 d for Herbert to 51 d for Paloma, with a mean duration of 26 d.

Table 2 shows the Pearson phenotypic correlation coefficients for the six phenological traits considered. Values of the correlation coefficient for trait pairs were significant and positive in most cases. Negative significant correlations were observed between flowering season (FS) and days to flowering (DF), flowering season (FS) and days to the end of flowering (DEF), and harvesting season (HS) and days to flowering (DF).

**Table 2:** Pearson correlation.

Phenological traits		DF	DEF	FS	DH	DEH	HS
Days to flowering	DF						
Days to the end of flowering	DEF	0.87***					
Flowering season	FS	-0.73***	-0.30*				
Days to harvesting	DH	0.48***	0.67***	ns			
Days to the end of harvesting	DEH	0.38**	0.63***	ns	0.94***		
Harvesting season	HS	-0.27*	ns	0.37**	ns	0.20*	

Pearson phenotypic correlation coefficients for six phenological traits using the mean data scored over six years. Correlation plus the significance level as stars are indicated (***, p < 0.001; **, 0.001> p <0.01; *, 0.01 > p < 0.05; ns, not significant).

### Genotyping

For the 70 cvs, sequencing of the GBS library yielded 272 million reads of good quality. The sequencing coverage was estimated to be 16x. After merging, the total number of tags was 1582133, with a minimum reads per tag of three. Of the tags, 25.8% were uniquely aligned and 185661 SNPs were identified. Data were filtered for MAF > 0.01 and no missing data per site, resulting in 42755 SNPs. Using this matrix, the error rate of the analysis was estimate to be 0.8% by comparing the duplicated DNA samples from Pilgrim. The 42755 SNPs were aligned to a total of 4258 scaffolds, ranging in size from 127 to 161211 bp, and including a minimum of one SNP per scaffold and a maximum of 65 SNPs per scaffold (64 bp max). In many cases, SNPs were located in the same tag which could overestimate the importance of these chromosome regions in genetic relationship analyses. Data were filtered in a second step for a minimum distance of 64 bp between adjacent SNPs, giving rise to a final matrix for genetic relationship analyses of 5255 SNPs distributed across 3917 scaffolds. Of these SNPs, 4280 showed a di-nucleotide variant, 240 showed a tri-nucleotide variant and 735 showed insertion-deletion points.

The heterozygocity proportion per taxa showed and average value of 0.22%, ranging from 0.02% of Pilgrim to 0.28% of PowderBlue (S2 Table). It can be expected the lowest heterozygocity rate in Pilgrim, the diploid *V*. *macrocarpum* cultivar. The highest heterozygocity levels were observed in the five rabbiteyes.

### UPGMA clustering

**Fig 2:**
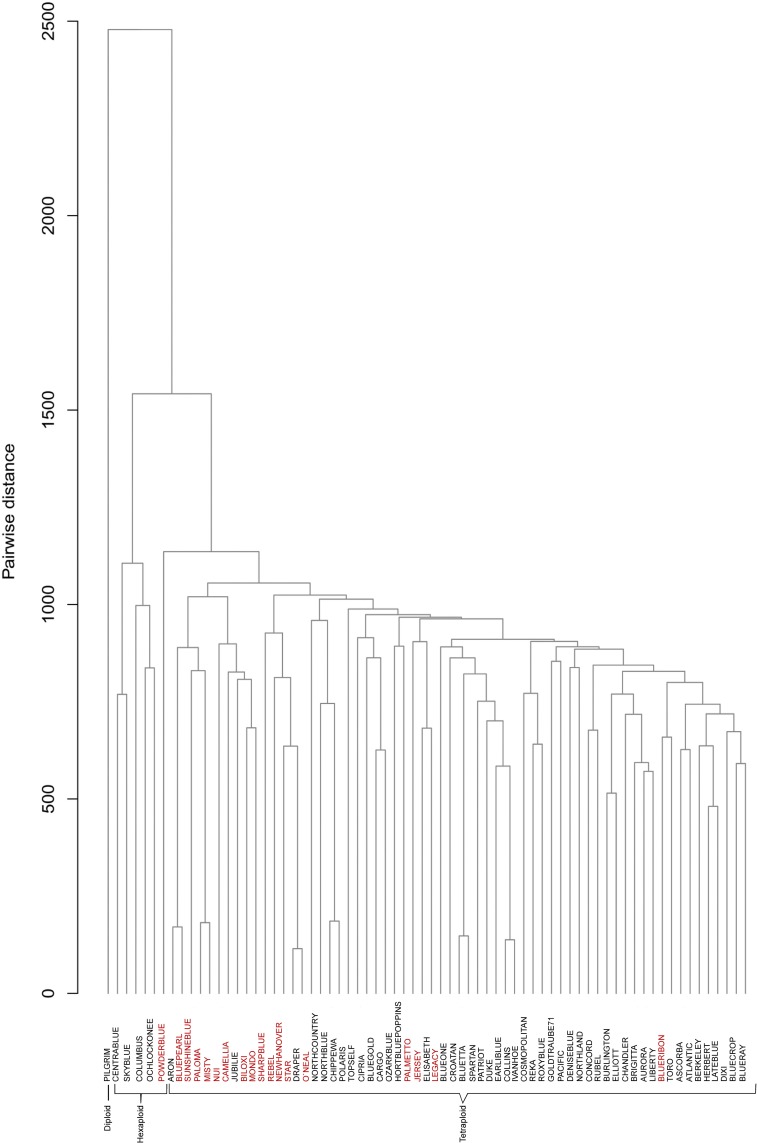
Cluster analysis. Phylogenetic relationship of the 70 Vaccinium spp accessions based on the analysis of 5255 SNPs obtained by GBS. Red color indicates varieties with low chill requirements under local growing conditions.

To establish phylogenetic relationships among the 70 cvs, a dendrogram was constructed using the UPGMA method and 5255 SNPs (Fig 2). Three main groups were observed, one including the diploid *V*. *macrocarpum* Pilgrim, the second including the five hexaploid *V*. *virgatum* cvs, and the third including the tetraploid species *V uliginosum* and *V*. *corymbosum*. In any group duplicated cvs were observed, although some pairs were very similar, with low values between them in the pairwise distance matrix (S3 Table): Bluepearl and Sunshine, Misty and Nui, Draper and O´Neal, Ivanhoe and Collins, and Bluetta and Spartan. Most of the 18 varieties classified as having low chilling requirements under local growing conditions were clustered into two groups: one group included eight cvs (Blue Pearl, Sunshineblue, Paloma, Misty, Nui, Camellia, Biloki, Mondo, and Sharpblue), while the other includes four (Rebel, Newhanover, Star, and O´Neal). Four of the five half-high cvs were clustered together (Northcountry, Northblue, Chipewa and Polaris).

### PCA of the *V*. *corymbosum* group

**Fig 3:**
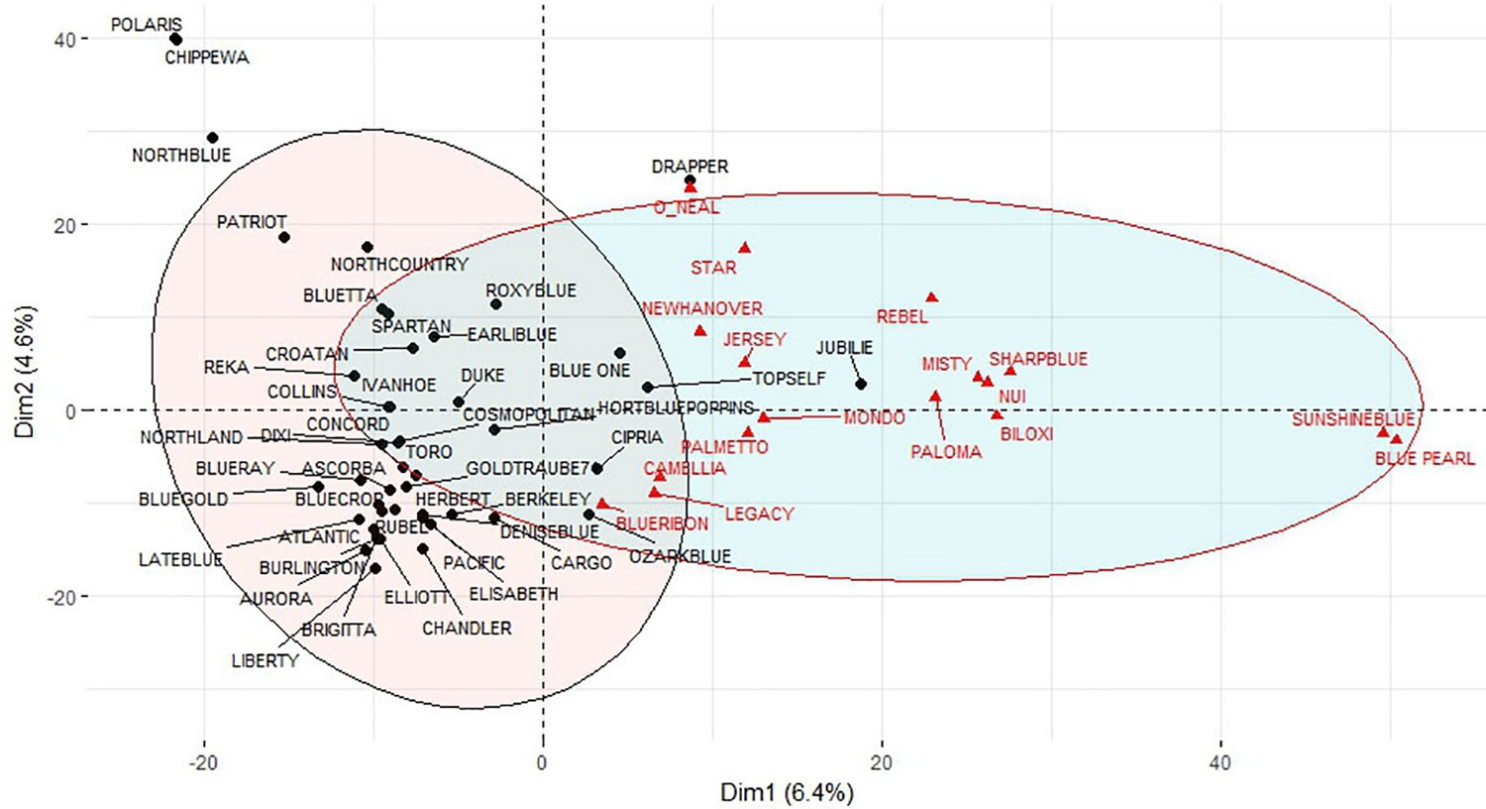
Principal component analysis. Two-dimension plot obtained from principal component analysis for 63 V. corymbosum varieties and data of 3614 SNPs. Confident ellipses are estimated by FactoMiner package in R with a threshold of 0.9. Red color indicates varieties with low chill requirements under local growing conditions.

A PCA analysis was conducted on the 63 *V*. *corymbosum* and half-high cvs in order to further define the genetic relationships within this group. Only the polymorphic SNPs within *V*. *corymbosum* were used (3614 SNPs). Fig 3 shows the two-dimensional plot obtained. The first and second components represent the 6.4% and 4.6% of the total variation, respectively. Two main groups were identified, one including mainly cvs with low-chilling requirement and other including varieties with high-chilling requirement. The five *V*. *corymbosum* cvs with mid-chilling requirement (Roxyblue, Earliblue, Blueone, Topself and Cipria) were located in a central position, at the intersection between the two groups. Polaris, Chippewa, NorthBlue, Drapper and O´Neal exhibited the highest values in the second dimension (Dim2) and their position within the plot was outside the distribution of the two main groups.

**Fig 4:**
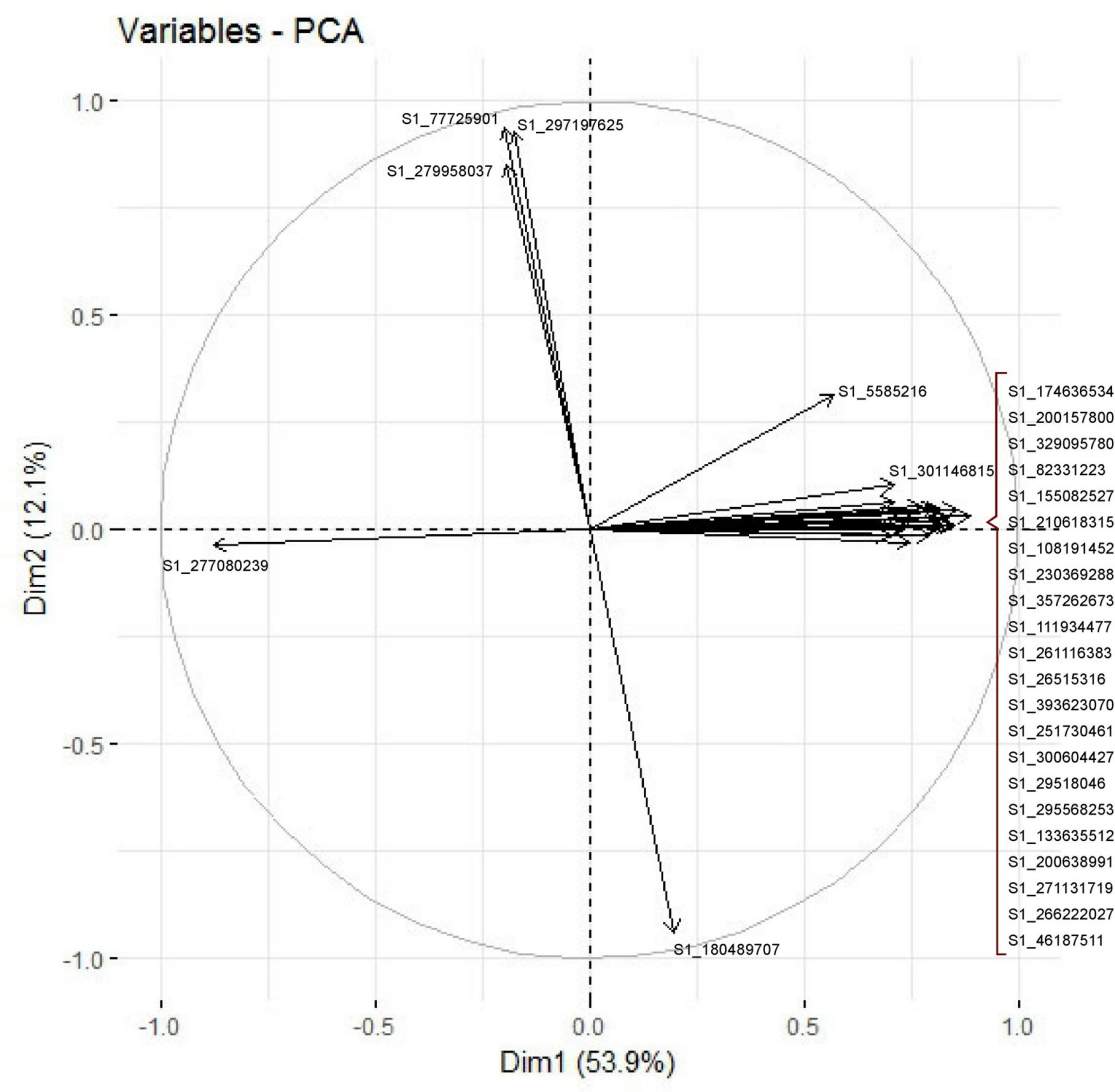
Most informative SNPs of the PCA. Name and x, y coordinates of the 29 SNPs showing the highest contribution to the Dim1 and Dim2 of the PCA.

Among the 3614 SNPs, 29 showed the greatest contribution to the Dim1 and Dim2 of the PCA, explaining altogether 66% of the total variation (Fig 4; S4 Table). The PCA obtained using the 29 SNPs (S1 Fig) was similar to that obtained with the 3614 SNPs (Fig 4), allowing a clear differentiation between cvs with low and high chilling requirements.

## Discussion

Commercial blueberry cvs have been grown in Europe most recently, since 1930s, so there is not much information available about their adaptation into new regions, although expansion of this crop depends to a large extent on the availability of cvs adapted to a broad spectrum of environments. One of the most important decisions for berry producers and for expansion of this crop is cv selection, but little information is available on their adaptation to different geographical areas, beyond tree nurseries. Generally, the characteristics of a cv are defined under environmental conditions in which it was obtained that may be quite different from those in the production area. Moreover, the changing global environment with higher global temperature and extreme fluctuations in rainfall patterns, make it important for breeders to know about the adaptation of genotypes to different environments [35]. In this work, we investigated the adaptation of a set of 70 blueberry cvs of American origin to the environmental conditions in northern Spain (Europe), and assessed the genetic diversity within this set using the SNP variation supplied by a GBS analysis.

Flowering season, associated with chilling requirements, and harvesting season are important traits for the adaptation of the cvs to different environments. Most of the cvs evaluated had high-chilling requirements so can be classified as northern cvs under local conditions. Cvs with low-chill requirements, classified as southern, can exhibit problems in this area because low morning temperatures, fog or late frosts and low populations of pollinating insects during February-March hinder the maturation process of the flowers in these early-flowering cvs. Some cvs showed a stable response over the years for these traits, such as Bluecrop or Elizabeth which showed low variation (error) between years for the initiation date of flowering or the initiation day of harvesting. The flowering or harvesting dates of other cvs, as Aron or Dixi, seemed to be more dependent on the weather conditions of each individual year, and showed a margin of error greater than 10 d among years. This result is in agreement with previous studies which suggested that a cv can show different chilling requirements based on the environmental conditions [36,37]. For this reason, some cvs can be difficult to classify, for example Aurora, Late Blue or Dixi, identified at first as mid-season cvs but could also be classified as late-season based on the margin of error. The most typical southern cvs, Star [15], O´Neal [38], Rebel, Camellia, Palmetto, Misty, Sharpblue or NewHanover [11], also behaved like southern cvs under local conditions in northern Spain. Aron (*V*. *uliginosum*) exhibited a high-chilling requirement and was the last cv to start flowering, as would be expected for a cv adapted to the hard climatic conditions of Finland [39]. With regard to harvesting season, this set of cvs allowed harvesting of fruit from the end of May to the end of September. Under local Spanish conditions, fruit ripening beyond October could present problems due to falling temperatures and a decrease in daylight hours. Most cvs were early or mid-season and a shortage of late season highbush cvs was identified. The latest harvested highbush cvs were Dixi, Aurora, Late Blue, and Burlington, from which harvest began around mid-July. Liberty and Elliott are regarded as the standard late season blueberry cvs in Europe, but in northern Spain were classified as mid-season cvs. Late and extra late cvs that produce fruits in September were rabbiteye types (CentraBlue, SkyBlue, Columbus, and Ochlockonee) which have a smaller, less tasty fruit than the *V*. *corymbosum* cvs. Harvest period has an important effect on crop yield, because market price fluctuates based on fruit availability, so off-peak market harvests are highly valued. Results obtained in this work revealed a lack of *V*. *corymbosum* cvs for late or extra-late season harvesting under local conditions.

For the development of new cvs and the planning of breeding programs, it is important to have detailed information on the available genetic diversity. In this study, the genetic diversity collected in a set of 70 diploid, tetraploid and hexaploid blueberry cvs was studied through GBS. Genotyping variants identified in polyploid species using bi-allelic markers such us SNPs, are less informative than in diploids because variants can be represented not only by a number of different allele combinations but also by different zygosity levels. For these reasons, GBS in polyploids requires greater read depths, at least 15x has been suggested for tetraploids compared to diploids [40]. In this study a read depth of 16x was obtained which allowed for a quite accurate genotyping of blueberry cvs. Based on the 5255 SNP markers obtained, cluster analyses revealed three main groups associated with the ploidy level of cvs: diploids cvs (*V*. *macrocarpum*), hexaploids (*V*. *virgatum*), and tetraploids (*V uliginosum* and *V*. *corymbosum*). This aggrupation related to ploidy level was also observed by Bian et al. [41] using only a set of 42 SSR markers.

The five hexaploid genotypes (rabbiteyes) appeared to be closely related according to the dendrogram. A narrow genetic base for rabbiteyes was evidenced using RAPD (random amplified polymorphic DNA) [42] and EST (expressed sequence tags) markers [26], which is to be expected as pedigree analysis suggests that most rabbiteyes have only four native selections of *V*. *virgatum* in their genetic background [43]. Also, some rabbiteyes share parental varieties in their genealogies, as Centrablue and Sky Blue that are selections derived from the cross Centurion x Rahi (see Table 1).

In the tetraploid group, *V*. *uliginosum* Aron [39] was clearly differentiated from *V*. *corymbosum* cvs. At first, a narrow genetic base of the *V*. *corymbosum* commercial cvs was expected, as the basis for the establishment of the modern blueberry industry was four genotypes selected by Dr F Coville in the first blueberry breeding programs [24,44]. Cluster analysis revealed that most of the southern cvs studied were closely related, which was also observed by Boches et al. [25] analyzing 69 accessions and 28 SSR markers. This result is consistent with the genetic basis, as southern varieties are hybrids between *V*. *corymbosum* highbushs and native species with lower chilling requirements [11,24,45]. Most cvs classified as southern blueberries appeared grouped in the dendrogram with Sharpblue, the first southern cv developed in 1966 [46]. This result suggests that the other southern cvs BluePearl, Sunshineblue, Paloma, Misty, Camellia, Biloxi, and Mondo may derive from the original Sharpblue. In fact, as far as we can know Camellia and Biloxi derive from Sharpblue in their pedigree (see Table 1). Another group of closely related southern cvs included Rebel, NewHanover, Star and O´Neal. This group probably derived from the oldest cv present, O´Neal, selected in 1972 [38]. Cvs O’Neal and Draper appear outside the two main groups obtained in the PCA analysis. This position agrees with the respective pedigrees; O´Neal has been reported to be the southern cv with the highest proportion of *V*. *angustifolium* genome, while Draper is composed primarily of genes of *V*. *corymbosum*, but also has contributions from three other species, *V*. *tenellum*, *V*. *ashei* and *V*. *darrowi* [45]. With respect to half-highs, all except Northland, appear to be genetically further removed from the remaining *V*. *corymbosum* cvs. This separation is in agreement with their genetic basis, as half-highs are hybrids between *V*. *corymbosum* and *V*. *angustifolium* [11,47]. Most of the half-high cvs used in this work are part of the same breeding program which included the cross G65 x Asworth in their pedigree [41,47], so a close relationship between them is to be expected.

Special mention should be made of Nui, a historically northern highbush cv which in this work appeared to be very similar to the southern cv Misty and which clustered closely to the other southern cvs. It also showed low chilling requirements under local conditions. It is possible that some problems of synonymy or homonymy exit between cvs in a crop like blueberry, which exhibits very little morphological variation in fruits or plants. It is not unusual for commercial growers and breeders to have difficulty distinguishing blueberry cvs. Proper cv identification is an important requisite in commercial plantings and breeding nurseries, and for optimization of germplasm maintenance and utilization. In this work, grouping the cvs according to their chilling requirements could be achieved using a set of only 29 SNP markers. These SNPs could have a practical application for breeding programmes focused on the agronomic trait chilling requirement, which is a key factor for the environmental adaptation of a species. The tag sequence in which these SNPs are included is provided in this work (S4 Table) and could be used to develop markers more applicable to routine laboratory screening, such as SCARs (Sequence Characterized Amplified Region), SSRs (Single Sequence Repeats) or InDels (Insertion/Deletion). A gene involved in flowering and chilling requirements, the *VcFT* gene, was isolated from *V*. *corymbosum* and located in the Vaccinium_corymbosum_v1_Contig341 (available at the Genome Database for Vaccinium www.vaccinium.org) [48]. This contig was aligned with the *V*. *macrocarpon* genome and was located in scaffold_27371 (e-value = 0.0; 95% identities). However, none of the 29 SNPs identified in this work were included in this scaffold (see S4 Table), suggesting that other chromosome regions, apart from that containing the *VcFT* gene, could be involved in flowering and chilling requirements in *Vaccinium*. Apart from this set of 29 markers, the remaining 5226 SNPs obtained in this work can be directly applied for blueberry marker assisted breeding, which is very important for crop species like blueberry lacking extensive genomic resources.

## Conclusions

The results from this research contribute useful knowledge on the adaptation of of a set of 70 blueberry cvs to local conditions of northern Spain, which can be translated to similar latitudes, and which revealed a shortage of late-harvested blueberry cvs. In addition, this work provided data on the genetic diversity within the set from a massive genotyping, which can be used in the preservation of genetic resources in this crop and in the planning of future plant breeding programs. To know the genetic distance between materials is very useful for breeders, whom should make conscious efforts to broaden the blueberry genetic base for the development of new blueberry cvs.

## Materials and methods

### Plant material

A field collection of *Vaccinium* spp is maintained at the SERIDA (Regional Service for Agri-Food Research and Development) station in Asturias, northern Spain (43º 29´ 01´´N, 5º 26´ 11´´W; elevation 6.5 m). Two plants per cv were spaced planted at 1 x 2.5 m and watered by drip irrigation. Each of the 70 cvs used was represented by plants which are at least three years old at the experimental period 2012–2017. The set includes 57 *V*. *corymbosum* cvs, five *V*. *virgatum*, one *V*. *macrocarpum*, one *V*. *uliginosum*, and six half-high hybrids (Table 1). Pedigree data on each cv are presented where available based on Moore [44], Trehane [12], Boches et al. [25], and information of the National Clonal Germplasm Repository (NCGR) of the United States Department of Agriculture (USDA). Most varieties were obtained from European plant nurseries, except Aron, Ascorba, Burlington, Concord, Croatan, Dixi, Jubilie, Pacific, and Premier that were obtained from the NCGR-USDA collection.

### Phenological traits

During the six years of the trials (2012 to 2017), the following phenological traits were collected relative to 1 January of each year: days to flowering (10% of the flowers are open), days to end of flowering, days to harvest beginning (25% of the fruits can be harvested), and days to the end of harvest (more than 90% of the fruits have been harvested). The two plants per cv were scored independently once each week during the flowering and ripening seasons and the average value was calculated per cv and per year. The flowering season was calculated as: (mean of days to flowering)–(mean of days to end of flowering). According to the date estimated for the initiation of flowering, chilling requirements were deduced for each cv and they were classified as low-chilling requirement (flowering before 1^st^ March), mid-chilling requirement (between 1 and 15 March) or high-chilling requirement (later than 15 March). The estimated number of chilling hours per year (threshold 7ºC) for this region of Spain is 834±143 h based on the daily temperature data stored at the National Bank of Climatological Data (AEMET) for the 2002–2012 periods (http://www.aemet.es/documentos/es/serviciosclimaticos/datosclimatologicos/atlas_climatico/Mapas_de_riesgo_2002-2012_WEB.pdf). The fruit season was calculated as: (mean of days to the start of harvesting)–(mean of days to the end of harvesting). Cvs were classified according to the date estimated for the start of harvest, from early season (harvest beginning < 15 June), mid season (between 16 June and 20 July), late season (between 21 July and 25 August) or extra-late season (> 25 August).

Pearson’s correlation coefficients among the phenological traits were investigated using the mean phenotypic data scored over six years. Statistical analyses were carried out using the PerformanceAnalytics package of the R project for statistical computing [49].

### DNA extraction

Young leaves from one plant of each cv were collected, frozen in liquid nitrogen and pulverized. DNA was extracted using the CTAB method described by Doyle and Doyle [50] and polivinilpirrolidona (PVP) 0.5 p/v was added to the buffer extraction to remove phenolic compounds. DNA was quantified photo metrically (260–280 nm) using a Biomate 3 ultraviolet–visible spectrophotometer (Thermo Scientific, Waltham, Massachusetts, USA). The quality of each DNA sample was verified in 1% agarose gels, stained with RedSafe (INtRON, Biotechnology, Gyunggi-Do, Korea) and visualized under ultraviolet light. Samples were storage at –80°C.

### Genotyping by sequencing

GBS analysis was carried out at the Institute of Genomic Diversity, Cornell University (Ithaca, NY, USA). DNAs were digested individually with the *ApeK*I restriction enzyme, which recognizes a five base pair sequence (GCWGC, where W can be either A or T). A GBS sequencing library was prepared by ligating the digested DNA to unique nucleotide adapters (barcodes) followed by PCR with flow-cell attachment site tagged primers. Sequencing was performed using Illumina HiSeq2000. The sequencing coverage obtained was estimated using the equation proposed by Lander and Waterman [51], C = LN/G, where, C is the stand for coverage, L is the read length, N is the number of reads and G is the genome length. Sequencing reads were aligned using the Burrow Wheelers Alignment tool V 0.7.8-r455 [52] to the diploid *V*. *macrocarpum* genome deposited in the National Center of Biotechnology (BioProject PRJNA245813) [29]. This was the only *Vaccinium* genome available when this work was conducted and it was used for its high degree of macro-synteny and collinearity with *V corymbosum* [53].

### Data analysis

SNP markers were collected using the GbS pipeline implemented in TASSEL 5.2.33 software [54]. Due to the complexity of genotype polyploid species with bi-allelic markers such as SNPs, all heterozygous classes in tetraploids (for example AAAa, AAaa, or Aaaa) were scored as the same (Aa). Data were filtered considering no missing values and minor allele frequency (MAF)> 0.01. Two duplicate DNA samples of cv Pilgrim derived from the same isolation process were included in the plate in order to estimate the error rate of this GBS analysis, calculated as the ratio of the differences between duplicated samples by the total number of SNPs.

A phylogenetic tree was built with the FactoMineR [55] package of R, using the pairwise distance and the UPGMA (Unweighted Pair Group Method with Arithmetic Mean) agglomeration method. FactoMineR package was also used to compute a Principal Component Analysis (PCA). The contribution of each SNP in the final PCA plot was visualized with the function fviz_contrib() of factoextra [56] package.

## Supporting information

S1 Fig PCA based on the most informative SNPs. Two-dimension plot obtained from principal component analysis for 63 varieties and the most 29 informative SNPs. Red color indicates varieties with low-chilling requirements.
(PDF)

S1 Table Raw data from phenological traits. Mean values obtained from 2012 to 2017 for days to flowering (10% of the flowers are open), days to end of flowering, days to harvest beginning (25% of the fruits can be harvested), and days to the end of harvesting (more than 90% of the fruits have been harvested). Duration of flowering and harvest season is indicated in days. Only plants with at least three years-old were considered for the characterization.
(PDF)

S2 Table Heterozygocity per taxa. Heterozygocity proportion per taxa estimated for 5255 SNPs.
(PDF)

S3 Table Distance matrix. Pairwise distance matrix obtained in R for 70 blueberry varieties and 5255 SNPs.
(PDF)

S4 Table Tag sequence of 29 selected SNPs. Tag sequence including the 29 most informative SNPs identified in the PCA analyses. The scaffold of the *V*. *macrocarpon* genome (available at www.vaccinium.org) which includes the tag sequence is indicated. The allele variation identified is indicated between brackets in red color (+, insertion; -, deletion).
(PDF)
