# Supplementary material for: Genetic diversity assessed by genotyping by sequencing (GBS) and for phenological traits in blueberry cultivars
Source: PLoS One. 2018 Oct 23;13(10):e0206361. doi: 10.1371/journal.pone.0206361 (PMC6198992; doi:10.1371/journal.pone.0206361)
Supplement: S1 Fig — Two-dimension plot obtained from principal component analysis for 63 varieties and the most 29 informative SNPs. Red color indicates varieties with low-chilling requirements. (PDF) [file pone.0206361.s001.pdf]

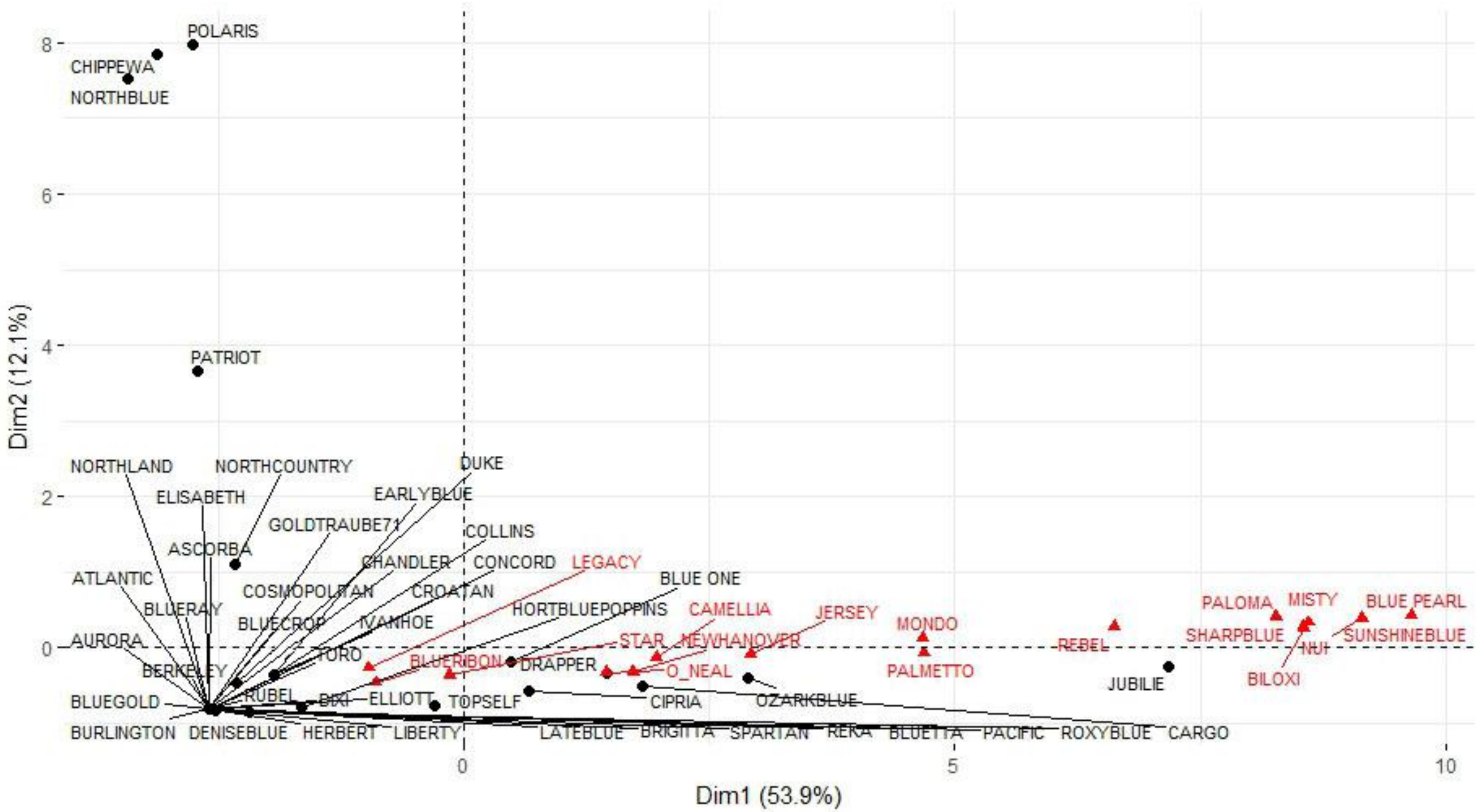

Fig. S1. Two-dimension plot obtained from principal component analysis for 63 varieties and the most 29 informative SNPs. Red color indicates varieties with low-chilling requirements.
