# Supplementary material for: Genetic diversity assessed by genotyping by sequencing (GBS) and for phenological traits in blueberry cultivars
Source: PLoS One. 2018 Oct 23;13(10):e0206361. doi: 10.1371/journal.pone.0206361 (PMC6198992; doi:10.1371/journal.pone.0206361)
Supplement: S1 Table — Mean values obtained from 2012 to 2017 for days to flowering (10% of the flowers are open), days to end of flowering, days to harvest beginning (25% of the fruits can be harvested), and days to the end of harvesting (more than 90% of the fruits have been harvested). Duration of flowering and harvest season is indicated in days. Only plants with at least three years-old were considered for the characterization. (PDF) [file pone.0206361.s002.pdf]

**Table S1.** Mean values obtained from 2012 to 2017 for days to flowering (10% of the flowers are open), days to end of flowering, days to harvest beginning (25% of the fruits can be harvested), and days to the end of harvesting (more than 90% of the fruits have been harvested). Duration of flowering and harvest season is indicated in days. Only plants with at least three years-old were considered for the characterization.

| Cultivar[a] | Days to flowering (± error) | Dats to end flowering (± error) | Flowering season (days) | Days to haverst beginning (± error) | Days to the end harvest (± error) | Harvest season (days) |
|---|---|---|---|---|---|---|
| Pilgrim* | - | - | | - | | - |
| Aron* | 118 ± 17.3 | 141 ± 11.5 | 22 | 181 ± 10.0 | 197 ± 12.0 | 16 |
| Centrablue | 98 ± 13.0 | 158 ± 26.9 | 60 | 257 ± 9.4 | 273 ± 4.3 | 15 |
| Columbus | 71 ± 6.5 | 132 ± 5.5 | 61 | 202 ± 5.2 | 242 ± 4.4 | 40 |
| Ochlockonee | 71 ± 6.3 | 129 ± 3.4 | 58 | 212 ± 2.7 | 248 ± 5.3 | 36 |
| Powderblue* | 57 ± 5.3 | 112 ± 0.8 | 55 | 198 ± 3.5 | 228 ± 3.6 | 30 |
| Sky Blue | 98 ± 12.0 | 137 ± 20.4 | 39 | 228 ± 7.5 | 247 ± 5.2 | 19 |
| Chippewa | 87 ± 5.4 | 131 ± 3.9 | 44 | 168 ± 3.6 | 200 ± 3.9 | 32 |
| Northblue* | 74 ± 6.7 | 115 ± 3.3 | 40 | 164 ± 2.7 | 187 ± 2.6 | 23 |
| Northcountry | 86 ± 10.8 | 118 ± 10.3 | 32 | 168 ± 7.4 | 186 ± 13.5 | 17 |
| Northland* | 65 ± 10.5 | 108 ± 2.9 | 43 | 162 ± 2.7 | 184 ± 2.0 | 23 |
| Polaris | 87 ± 10.1 | 126 ± 10.0 | 38 | 171 ± 5.7 | 191 ± 5.2 | 20 |
| Sunshine Blue | 57 ± 16.4 | 91 ± 14.9 | 35 | 182 ± - | 201 ± - | 19 |
| Ascorba | 91 ± 10.8 | 113 ± 15.5 | 22 | 177 ± 10.5 | 195 ± 14.5 | 18 |
| Atlantic* | 97 ± 7.8 | 125 ± 5.5 | 28 | 177 ± 2.9 | 210 ± 4.6 | 34 |
| Aurora | 103 ± 5.9 | 136 ± 3.6 | 33 | 198 ± 4.1 | 231 ± 6.2 | 33 |
| Berkeley* | 90 ± 5.9 | 122 ± 4.2 | 32 | 174 ± 3.1 | 203 ± 4.2 | 29 |
| Biloxi | 31 ± 4.2 | 89 ± 8.5 | 58 | 167 ± 3.7 | 200 ± 3.8 | 33 |
| Blue One | 62 ± 7.9 | 119 ± 4.1 | 57 | 161 ± 3.4 | 194 ± 12.5 | 33 |
| Blue Pearl | 32 ± 8.0 | 98 ± 8.7 | 66 | 178 ± 4.7 | 206 ± 3.2 | 28 |
| Blue Ribon | 53 ± 14.2 | 88 ± 20.5 | 34 | 171 ± 6.0 | 196 ± 5.5 | 25 |
| Bluecrop* | 92 ± 3.7 | 126 ± 5.3 | 34 | 175 ± 2.7 | 204 ± 3.5 | 30 |
| Bluegold | 87 ± 5.4 | 120 ± 3.4 | 34 | 174 ± 2.9 | 198 ± 4.5 | 24 |
| Blueray | 97 ± 6.0 | 127 ± 3.1 | 30 | 172 ± 2.2 | 200 ± 3.1 | 27 |
| Bluetta* | 83 ± 5.1 | 114 ± 3.2 | 30 | 160 ± 0.9 | 185 ± 1.0 | 25 |
| Brigitta | 95 ± 6.7 | 129 ± 2.9 | 34 | 184 ± 4.2 | 210 ± 3.0 | 26 |
| Burlington* | 109 ± 10.3 | 129 ± 10.1 | 20 | 197 ± 7.6 | 211 ± 4.9 | 15 |
| Camellia | 35 ± 4.6 | 94 ± 8.8 | 59 | 171 ± 2.2 | 193 ± 2.7 | 22 |
| Cargo | 81 ± 5.4 | 114 ± 3.2 | 34 | 180 ± 4.4 | 201 ± 5.0 | 21 |
| Chandler | 78 ± 5.0 | 119 ± 3.2 | 42 | 175 ± 6.4 | 204 ± 4.2 | 29 |
| Cipria | 74 ± 13.3 | 111 ± 17.4 | 37 | 168 ± 4.8 | 198 ± 5.9 | 31 |
| Collins* | 82 ± 4.1 | 116 ± 3.4 | 34 | 165 ± 2.3 | 187 ± 1.6 | 22 |
| Concord* | 112 ± 10.9 | 143 ± 3.3 | 31 | 182 ± 9.5 | 208 ± 2.9 | 26 |
| Cosmopolitan | 97 ± 7.5 | 127 ± 4.3 | 30 | 177 ± 3.4 | 202 ± 3.5 | 26 |
| Croatan* | 87 ± 7.1 | 111 ± 5.8 | 24 | 172 ± 5.5 | 191 ± 10.5 | 19 |
| Denise Blue | 78 ± 9.0 | 125 ± 3.3 | 47 | 179 ± 4.7 | 211 ± 2.5 | 32 |
| Dixi* | 110 ± 11.8 | 143 ± 3.3 | 33 | 197 ± 12.0 | 222 ± 8.0 | 25 |
| Draper | 92 ± 6.5 | 124 ± 3.2 | 32 | 168 ± 1.8 | 190 ± 7.4 | 22 |
| Duke* | 93 ± 6.1 | 122 ± 2.5 | 30 | 163 ± 2.7 | 182 ± 2.8 | 18 |
| Earliblue* | 70 ± 7.1 | 113 ± 1.5 | 43 | 157 ± 3.2 | 181 ± 3.1 | 24 |
| Elizabeth* | 92 ± 2.9 | 127 ± 3.9 | 35 | 188 ± 5.1 | 222 ± 4.5 | 34 |
| Elliott* | 103 ± 8.0 | 132 ± 4.3 | 29 | 192 ± 4.3 | 217 ± 6.3 | 26 |
| Goldtraube 71 | 96 ± 7.1 | 132 ± 6.2 | 36 | 175 ± 4.3 | 203 ± 5.3 | 29 |
| Herbert* | 113 ± 11.2 | 132 ± 7.2 | 19 | 181 ± - | 195 ± - | 14 |
| Hortblue Poppins | 77 ± 7.4 | 114 ± 4.2 | 37 | 171 ± 3.2 | 194 ± 2.3 | 23 |
| Ivanhoe* | 100 ± 4.6 | 129 ± 3.1 | 29 | 172 ± 3 | 200 ± 1.9 | 28 |
| Jersey* | 54 ± 19.2 | 75 ± 19.5 | 21 | 167 ± 0.5 | 185 ± - | 19 |
| Jubilie | 86 ± 8.3 | 108 ± 3.5 | 22 | 166 ± 6.5 | 188 ± 5.8 | 23 |
| Late Blue | 112 ± 7.1 | 135 ± 4.1 | 23 | 198 ± 3.0 | 219 ± 3.4 | 20 |

**Table S1**.Continued

| Cultivar[a] | Days to flowering (± error) | Dats to end flowering (± error) | Flowering season (days) | Days to haverst beginning (± error) | Days to the end harvest (± error) | Harvest season (days) |
|---|---|---|---|---|---|---|
| Legacy | 46 ± 6.5 | 101 ± 6.4 | 55 | 171 ± 2.3 | 199 ± 2.5 | 28 |
| Liberty | 94 ± 5.0 | 129 ± 3.7 | 35 | 188 ± 4.3 | 216 ± 4.2 | 28 |
| Misty | 30 ± 3.7 | 85 ± 8.1 | 55 | 155 ± 2.4 | 184 ± 2.8 | 29 |
| Mondo | 59 ± 10.1 | 106 ± 6.4 | 47 | 180 ± 3.1 | 208 ± 8.6 | 28 |
| New Hanover | 51 ± 19.8 | 79 ± 17.6 | 28 | 155 ± 12.0 | 172 ± - | 17 |
| Nui | 26 ± 3.0 | 87 ± 10.7 | 61 | 155 ± 2.3 | 181 ± 1.5 | 26 |
| O´Neal* | 41 ± 6.4 | 104 ± 5.2 | 63 | 165 ± 2.8 | 193 ± 2.6 | 28 |
| Ozarkblue | 87 ± 7.9 | 124 ± 2.5 | 37 | 185 ± 4.7 | 214 ± 2.4 | 28 |
| Pacific* | 99 ± 6.8 | 136 ± 10.0 | 37 | 179 ± 8.0 | 206 ± 14.0 | 27 |
| Palmetto | 34 ± 7.0 | 95 ± 9.8 | 61 | 159 ± 3.6 | 181 ± 3.6 | 22 |
| Paloma | 53 ± 19.4 | 82 ± 15.5 | 29 | 143 ± - | 194 ± - | 51 |
| Patriot | 78 ± 5.5 | 105 ± 2.9 | 27 | 168 ± 4.8 | 182 ± 1.6 | 15 |
| Rebel | 24 ± 3.5 | 88 ± 8.7 | 64 | 152 ± 3.5 | 182 ± 2.8 | 31 |
| Reka* | 79 ± 6.3 | 116 ± 2.6 | 37 | 162 ± 4.0 | 187 ± 4.0 | 25 |
| Roxy Blue | 61 ± 8.8 | 114 ± 3.8 | 53 | 167 ± 3.6 | 195 ± 1.5 | 28 |
| Rubel* | 92 ± 6.2 | 126 ± 3.0 | 34 | 172 ± 2.0 | 196 ± 2.7 | 24 |
| Sharpblue* | 20 ± 5.3 | 86 ± 8.1 | 66 | 159 ± 2.2 | 185 ± 2.6 | 27 |
| Spartan* | 87 ± 7.0 | 111 ± 2.4 | 25 | 161 ± 2.9 | 182 ± 3.0 | 20 |
| Star | 37 ± 6.8 | 88 ± 7.9 | 51 | 151 ± 2.6 | 183 ± 2.4 | 32 |
| Topself | 70 ± 14.7 | 114 ± 3.2 | 44 | 175 ± 6.0 | 197 ± 6.2 | 21 |
| Toro | 87 ± 6.0 | 123 ± 3.1 | 36 | 171 ± 3.9 | 200 ± 2.7 | 29 |

[a]Asterisk in superscript indicate accessions included in the NCGR-USDA *Vaccinium* Core Collection
