# Supplementary material for: Genetic diversity assessed by genotyping by sequencing (GBS) and for phenological traits in blueberry cultivars
Source: PLoS One. 2018 Oct 23;13(10):e0206361. doi: 10.1371/journal.pone.0206361 (PMC6198992; doi:10.1371/journal.pone.0206361)
Supplement: S2 Table — Heterozygocity proportion per taxa estimated for 5255 SNPs. (PDF) [file pone.0206361.s003.pdf]

Table S2. Heterozygocity proportion per taxa estimated for 5255 SNPs

Heterozygocity proportion	Taxa
0.02	Pilgrim
0.21	Liberty, Ascorba, Elliot, Toro, Dixi, Pacific, Goldtrabue71
0.22	Blueray, Cosmopolitan, Atlantic, Northcountry, Chandler, LateBlue, Herbert, Elizabeth, Camellia, Denise Blue, Polaris, Star, Berkeley, Brigitta, Croatan, Chippewa, Reka, Jersey, Aurora, Rubel, Aron, Bluecrop, Northblue, Duke, Ivanhoe, Blueone, Mondo, Burlington, Roxyblue
0.23	Horblue Poppins, Newhanover, Cipria, Concord, Northland, Rebel, Blueribon, Paloma, Biloki, Collins, Earliblue, Cargo, Palmetto, Patriot, Topself, Bluegold, Bluetta, Jubilie, Sharpblue, O´Neal, Draper, Spartan
0.24	Nui, Sunshineblue, Legacy, Misty, Ozarkblue, Bluepearl
0.25	Centrablue
0.26	Columbus
0.27	Ochlockonee, Skyblue
0.28	Powderblue
