# Supplementary material for: Genetic diversity assessed by genotyping by sequencing (GBS) and for phenological traits in blueberry cultivars
Source: PLoS One. 2018 Oct 23;13(10):e0206361. doi: 10.1371/journal.pone.0206361 (PMC6198992; doi:10.1371/journal.pone.0206361)
Supplement: S3 Table — Pairwise distance matrix obtained in R for 70 blueberry varieties and 5255 SNPs. (PDF) [file pone.0206361.s004.pdf]

# Table S4. Pairwise distance matrix obtained in R for 70 blueberry varieties and 5255 SNPs.

| | ARON | ASCORBA | ATLANTIC | AURORA | BERKELEY | BILOKI | BLUECROP | BLUEGOLD | BLUEONE | BLUEPEARL | BLUERAY | BLUERIBON | BLUETTA | BRIGITTA | BURLINGTON | CAMELLIA | CARGO | CHANDLER | CHIPPEWA | CIPRIA | COLLINS | CONCORD | COSMOPOLITAN | CROATAN | DENISEBLUE |
|---|---|---|---|---|---|---|---|---|---|---|---|---|---|---|---|---|---|---|---|---|---|---|---|---|---|
| ASCORBA | 1109 | | | | | | | | | | | | | | | | | | | | | | | | |
| ATLANTIC | 1113 | 627 | | | | | | | | | | | | | | | | | | | | | | | |
| AURORA | 1067 | 826 | 837 | | | | | | | | | | | | | | | | | | | | | | |
| BERKELEY | 1089 | 714 | 795 | 731 | | | | | | | | | | | | | | | | | | | | | |
| BILOKI | 1271 | 1109 | 1106 | 1106 | 1083 | | | | | | | | | | | | | | | | | | | | |
| BLUECROP | 1136 | 760 | 783 | 837 | 786 | 1055 | | | | | | | | | | | | | | | | | | | |
| BLUEGOLD | 1208 | 981 | 958 | 850 | 845 | 1157 | 782 | | | | | | | | | | | | | | | | | | |
| BLUEONE | 1184 | 949 | 1015 | 992 | 932 | 1105 | 922 | 1054 | | | | | | | | | | | | | | | | | |
| BLUEPEARL | 1282 | 1162 | 1144 | 1116 | 1021 | 1074 | 1144 | 1216 | 1120 | | | | | | | | | | | | | | | | |
| BLUERAY | 1077 | 783 | 796 | 851 | 810 | 1120 | 591 | 884 | 918 | 1162 | | | | | | | | | | | | | | | |
| BLUERIBON | 1168 | 917 | 941 | 957 | 863 | 1101 | 807 | 958 | 1004 | 1088 | 894 | | | | | | | | | | | | | | |
| BLUETTA | 1145 | 970 | 983 | 952 | 860 | 1130 | 892 | 979 | 917 | 1189 | 891 | 1006 | | | | | | | | | | | | | |
| BRIGITTA | 1107 | 921 | 942 | 588 | 751 | 1090 | 829 | 776 | 1073 | 1117 | 939 | 974 | 973 | | | | | | | | | | | | |
| BURLINGTON | 1022 | 892 | 896 | 719 | 790 | 1115 | 841 | 910 | 1054 | 1113 | 826 | 960 | 977 | 890 | | | | | | | | | | | |
| CAMELLIA | 1153 | 963 | 946 | 886 | 842 | 966 | 829 | 1023 | 1033 | 1053 | 881 | 952 | 1017 | 954 | 949 | | | | | | | | | | |
| CARGO | 1196 | 1009 | 942 | 976 | 934 | 1011 | 829 | 708 | 1096 | 1164 | 859 | 1007 | 1032 | 924 | 914 | 917 | | | | | | | | | |
| CHANDLER | 1050 | 828 | 840 | 729 | 676 | 1049 | 701 | 900 | 985 | 1052 | 738 | 863 | 902 | 706 | 844 | 866 | 911 | | | | | | | | |
| CHIPPEWA | 1154 | 993 | 986 | 997 | 1035 | 1196 | 935 | 1015 | 1086 | 1264 | 952 | 1093 | 1034 | 1033 | 1022 | 1045 | 1054 | 1049 | | | | | | | |
| CIPRIA | 1167 | 1041 | 989 | 998 | 986 | 1052 | 830 | 982 | 1053 | 1168 | 931 | 963 | 1062 | 1026 | 1005 | 945 | 927 | 905 | 1092 | | | | | | |
| COLLINS | 1033 | 847 | 928 | 852 | 757 | 1113 | 827 | 951 | 871 | 1109 | 829 | 940 | 866 | 907 | 834 | 966 | 981 | 851 | 954 | 1035 | | | | | |
| CONCORD | 762 | 900 | 874 | 849 | 877 | 1150 | 951 | 987 | 996 | 1126 | 875 | 999 | 975 | 906 | 776 | 992 | 1025 | 870 | 1036 | 1032 | 756 | | | | |
| COSMOPOLITAN | 1115 | 971 | 976 | 874 | 906 | 1121 | 679 | 851 | 1024 | 1160 | 806 | 943 | 950 | 847 | 879 | 938 | 889 | 756 | 973 | 985 | 891 | 966 | | | |
| CROATAN | 1084 | 908 | 1005 | 974 | 905 | 1148 | 937 | 994 | 977 | 1232 | 907 | 1031 | 916 | 1001 | 959 | 1050 | 1093 | 938 | 1093 | 1087 | 829 | 891 | 943 | | |
| DENISEBLUE | 1105 | 946 | 960 | 827 | 773 | 1137 | 858 | 877 | 1033 | 1110 | 866 | 977 | 997 | 725 | 912 | 930 | 945 | 777 | 1038 | 972 | 954 | 921 | 899 | 1045 | |
| DIXI | 1078 | 699 | 765 | 773 | 705 | 1062 | 672 | 924 | 891 | 1083 | 674 | 876 | 843 | 898 | 790 | 845 | 927 | 754 | 855 | 964 | 770 | 825 | 801 | 933 | 890 |
| DRAPPER | 1131 | 995 | 1065 | 1031 | 972 | 1108 | 1020 | 1128 | 942 | 1100 | 985 | 1081 | 938 | 1057 | 1020 | 1084 | 1114 | 1027 | 1089 | 1123 | 886 | 953 | 1016 | 906 | 1075 |
| DUKE | 1104 | 838 | 860 | 895 | 746 | 1099 | 809 | 920 | 861 | 1109 | 853 | 881 | 802 | 945 | 871 | 954 | 999 | 902 | 952 | 1013 | 747 | 896 | 868 | 875 | 909 |
| EARLIBLUE | 1064 | 830 | 959 | 889 | 796 | 1082 | 833 | 941 | 767 | 1135 | 847 | 920 | 693 | 943 | 898 | 985 | 981 | 927 | 950 | 1026 | 569 | 824 | 874 | 776 | 967 |
| ELISABETH | 1076 | 833 | 775 | 870 | 870 | 1147 | 861 | 937 | 1041 | 1197 | 880 | 857 | 1022 | 918 | 829 | 969 | 978 | 880 | 1034 | 1000 | 877 | 853 | 909 | 976 | 955 |
| ELLIOTT | 1040 | 835 | 781 | 578 | 765 | 1105 | 862 | 927 | 1036 | 1113 | 835 | 983 | 954 | 937 | 515 | 909 | 967 | 832 | 991 | 995 | 828 | 838 | 908 | 952 | 942 |
| GOLDTRAUBE71 | 1057 | 947 | 903 | 861 | 829 | 1131 | 896 | 998 | 1010 | 1141 | 867 | 992 | 953 | 936 | 873 | 1007 | 1025 | 882 | 1065 | 1021 | 918 | 850 | 923 | 977 | 957 |
| HERBERT | 1028 | 711 | 763 | 796 | 638 | 1114 | 727 | 924 | 912 | 1082 | 751 | 886 | 918 | 897 | 768 | 923 | 941 | 804 | 996 | 960 | 778 | 780 | 913 | 931 | 844 |
| HORTBLUEPOPPINS | 1158 | 873 | 909 | 983 | 785 | 1112 | 861 | 965 | 1043 | 1129 | 937 | 909 | 989 | 1002 | 979 | 985 | 1022 | 931 | 1053 | 1028 | 966 | 959 | 1016 | 966 | 941 |
| IVANHOE | 1032 | 852 | 937 | 847 | 751 | 1121 | 827 | 953 | 883 | 1102 | 816 | 925 | 876 | 900 | 834 | 958 | 969 | 846 | 950 | 1026 | 138 | 777 | 881 | 822 | 935 |
| JERSEY | 1167 | 1027 | 1052 | 1006 | 963 | 1038 | 994 | 1086 | 977 | 1072 | 997 | 989 | 1051 | 1027 | 999 | 1013 | 1082 | 985 | 1085 | 991 | 1003 | 981 | 1031 | 1002 | 1063 |
| JUBILIE | 1285 | 1043 | 1074 | 1083 | 1015 | 857 | 917 | 1073 | 1090 | 1137 | 1059 | 974 | 1083 | 1087 | 1123 | 975 | 1032 | 1043 | 1151 | 1072 | 1045 | 1114 | 982 | 1066 | 1103 |
| LATEBLUE | 1039 | 670 | 686 | 737 | 635 | 1122 | 663 | 886 | 947 | 1125 | 691 | 841 | 875 | 816 | 772 | 889 | 918 | 770 | 976 | 960 | 769 | 767 | 893 | 944 | 841 |
| LEGACY | 1169 | 945 | 922 | 1002 | 966 | 1084 | 854 | 1051 | 1082 | 1116 | 933 | 838 | 1082 | 1032 | 975 | 956 | 980 | 915 | 1085 | 937 | 982 | 970 | 1005 | 1056 | 1018 |
| LIBERTY | 1087 | 894 | 871 | 571 | 773 | 1069 | 876 | 872 | 1045 | 1101 | 888 | 992 | 994 | 599 | 738 | 921 | 969 | 718 | 1045 | 1041 | 915 | 907 | 905 | 1008 | 820 |
| MISTY | 1248 | 1035 | 1061 | 1035 | 897 | 1045 | 1027 | 1088 | 997 | 902 | 1068 | 1014 | 1075 | 1085 | 1065 | 1047 | 1099 | 1033 | 1147 | 1061 | 1037 | 1064 | 1043 | 1127 | 1025 |
| MONDO | 1176 | 956 | 975 | 995 | 916 | 908 | 890 | 1058 | 991 | 1040 | 879 | 1007 | 999 | 1002 | 1007 | 787 | 1030 | 927 | 1054 | 1015 | 954 | 981 | 978 | 1041 | 943 |
| NEWHANOVER | 1180 | 1043 | 1021 | 1022 | 1004 | 1104 | 959 | 1052 | 1031 | 1110 | 1012 | 961 | 1030 | 1070 | 1045 | 1007 | 1108 | 1028 | 1108 | 1024 | 982 | 1037 | 993 | 993 | 1094 |
| NORTHBLUE | 1184 | 964 | 1003 | 1015 | 987 | 1220 | 1006 | 1028 | 1036 | 1275 | 969 | 1134 | 992 | 1050 | 1020 | 1075 | 1056 | 996 | 745 | 1106 | 979 | 989 | 972 | 1056 | 981 |
| NORTHCOUNTRY | 1165 | 1054 | 1018 | 1007 | 994 | 1170 | 1007 | 982 | 1037 | 1223 | 977 | 1133 | 962 | 1018 | 1040 | 1052 | 1060 | 1037 | 975 | 1093 | 1010 | 1024 | 985 | 960 | 1035 |
| NORTHLAND | 1158 | 954 | 1040 | 878 | 672 | 1188 | 890 | 895 | 1070 | 1159 | 922 | 988 | 980 | 813 | 895 | 1006 | 1006 | 858 | 1037 | 1056 | 866 | 965 | 942 | 981 | 838 |
| NUI | 1250 | 1034 | 1071 | 1037 | 885 | 1010 | 1010 | 1085 | 988 | 878 | 1055 | 1002 | 1059 | 1069 | 1057 | 1005 | 1077 | 1025 | 1130 | 1040 | 1021 | 1071 | 1031 | 1108 | 1008 |
| OZARKBLUE | 1207 | 994 | 938 | 991 | 976 | 941 | 769 | 1018 | 1085 | 1132 | 846 | 997 | 1031 | 980 | 959 | 848 | 626 | 838 | 1054 | 835 | 976 | 1035 | 919 | 1138 | 983 |
| O_NEAL | 1139 | 992 | 1056 | 1037 | 974 | 1097 | 1023 | 1128 | 944 | 1104 | 991 | 1082 | 945 | 1054 | 1009 | 1076 | 1123 | 1033 | 1099 | 1119 | 878 | 940 | 1022 | 906 | 1066 |
| PACIFIC | 1129 | 918 | 843 | 904 | 809 | 1144 | 850 | 981 | 1071 | 1110 | 860 | 963 | 992 | 889 | 887 | 992 | 1010 | 849 | 1061 | 1014 | 974 | 972 | 898 | 1052 | 920 |
| PALMETTO | 1212 | 944 | 1056 | 1000 | 884 | 1000 | 952 | 1054 | 990 | 1018 | 977 | 928 | 1029 | 1046 | 1033 | 931 | 1044 | 969 | 1090 | 1048 | 933 | 1030 | 1053 | 1051 | 1064 |
| PALOMA | 1212 | 1020 | 1045 | 1029 | 888 | 920 | 1022 | 1092 | 1067 | 888 | 1053 | 1024 | 1048 | 1049 | 1017 | 991 | 1089 | 988 | 1127 | 1081 | 976 | 1006 | 1036 | 1113 | 1039 |
| PATRIOT | 1115 | 907 | 1004 | 951 | 907 | 1170 | 911 | 1024 | 930 | 1225 | 931 | 1066 | 895 | 1008 | 943 | 1049 | 1046 | 974 | 832 | 1082 | 773 | 890 | 986 | 921 | 982 |
| PILGRIM | 2299 | 2418 | 2421 | 2451 | 2447 | 2492 | 2448 | 2499 | 2474 | 2565 | 2436 | 2480 | 2500 | 2439 | 2470 | 2450 | 2496 | 2433 | 2454 | 2452 | 2488 | 2469 | 2434 | 2447 | 2431 |
| POLARIS | 1159 | 985 | 984 | 1001 | 1046 | 1204 | 935 | 1037 | 1077 | 1281 | 953 | 1107 | 1028 | 1047 | 1014 | 1041 | 1050 | 1048 | 186 | 1099 | 953 | 1016 | 957 | 1076 | 1031 |
| REBEL | 1225 | 1063 | 1090 | 1062 | 1011 | 958 | 1069 | 1175 | 1011 | 986 | 1062 | 1104 | 1036 | 1096 | 1113 | 1016 | 1140 | 1042 | 1142 | 1116 | 1037 | 1066 | 1091 | 1045 | 1086 |
| REKA | 1155 | 888 | 932 | 930 | 880 | 1137 | 663 | 889 | 993 | 1188 | 843 | 923 | 930 | 952 | 969 | 965 | 961 | 879 | 932 | 937 | 861 | 949 | 702 | 969 | 951 |
| ROXYBLUE | 1129 | 883 | 932 | 925 | 933 | 1091 | 802 | 966 | 990 | 1183 | 846 | 941 | 927 | 1008 | 995 | 1026 | 1005 | 907 | 993 | 989 | 897 | 965 | 841 | 921 | 996 |
| RUBEL | 990 | 797 | 712 | 867 | 806 | 1140 | 852 | 980 | 982 | 1128 | 839 | 1003 | 955 | 950 | 695 | 974 | 995 | 851 | 1036 | 1042 | 759 | 677 | 976 | 912 | 950 |
| SHARPBLUE | 1289 | 1083 | 1107 | 1038 | 1024 | 707 | 1092 | 1190 | 1061 | 1019 | 1134 | 1123 | 1085 | 1086 | 1101 | 867 | 1124 | 1047 | 1147 | 1111 | 1039 | 1060 | 1106 | 1127 | 1107 |
| SPARTAN | 1155 | 962 | 983 | 952 | 871 | 1114 | 889 | 983 | 922 | 1174 | 902 | 1001 | 148 | 984 | 977 | 996 | 1001 | 908 | 1031 | 1062 | 858 | 981 | 932 | 901 | 997 |
| STAR | 1186 | 1012 | 1065 | 1050 | 916 | 1107 | 1047 | 1116 | 962 | 1063 | 1021 | 1032 | 1013 | 1041 | 1044 | 1060 | 1105 | 1046 | 1083 | 1088 | 932 | 1047 | 1045 | 1013 | 1084 |
| SUNSHINEBLUE | 1280 | 1157 | 1145 | 1119 | 1016 | 1063 | 1141 | 1206 | 1123 | 171 | 1152 | 1078 | 1182 | 1103 | 1103 | 1043 | 1157 | 1060 | 1244 | 1140 | 1097 | 1122 | 1151 | 1209 | 1101 |
| TOPSELF | 1208 | 1007 | 995 | 1008 | 919 | 1090 | 962 | 1051 | 985 | 1047 | 955 | 977 | 1010 | 1037 | 968 | 1003 | 1032 | 980 | 1072 | 1004 | 939 | 1006 | 997 | 1070 | 1012 |
| CENTRABLUE | 1586 | 1531 | 1497 | 1522 | 1548 | 1503 | 1520 | 1549 | 1528 | 1484 | 1526 | 1550 | 1552 | 1527 | 1534 | 1509 | 1501 | 1517 | 1523 | 1521 | 1521 | 1501 | 1492 | 1523 | 1531 |
| COLUMBUS | 1589 | 1532 | 1507 | 1532 | 1566 | 1531 | 1533 | 1538 | 1523 | 1489 | 1507 | 1563 | 1531 | 1543 | 1520 | 1500 | 1491 | 1538 | 1515 | 1485 | 1542 | 1511 | 1554 | 1554 | 1494 |
| OCHLOCKONEE | 1617 | 1565 | 1528 | 1553 | 1562 | 1504 | 1564 | 1570 | 1529 | 1488 | 1537 | 1556 | 1571 | 1560 | 1539 | 1525 | 1498 | 1529 | 1548 | 1538 | 1557 | 1541 | 1558 | 1572 | 1573 |
| POWDERBLUE | 1624 | 1608 | 1562 | 1602 | 1617 | 1535 | 1598 | 1608 | 1560 | 1501 | 1587 | 1591 | 1604 | 1587 | 1579 | 1578 | 1549 | 1589 | 1595 | 1560 | 1584 | 1557 | 1603 | 1590 | 1592 |
| SKYBLUE | 1578 | 1568 | 1520 | 1565 | 1569 | 1514 | 1539 | 1585 | 1546 | 1505 | 1556 | 1579 | 1588 | 1574 | 1555 | 1516 | 1543 | 1548 | 1570 | 1510 | 1563 | 1540 | 1549 | 1546 | 1551 |
| TORO | 1092 | 776 | 786 | 781 | 682 | 1067 | 632 | 855 | 896 | 1100 | 775 | 659 | 820 | 790 | 858 | 883 | 944 | 713 | 974 | 902 | 799 | 885 | 819 | 878 | 860 |

| | DIXI | DRAPPER | DUKE | EARLIBLUE | ELISABETH | ELLIOTT | GOLDTRAUBE71 | HERBERT | HORTBLUEPOPPINS | IVANHOE | JERSEY | JUBILIE | LATEBLUE | LEGACY | LIBERTY | MISTY | MONDO | NEWHANOVER | NORTHBLUE | NORTHCOUNTRY | NORTHLAND | NUI | OZARKBLUE | O_NEAL | PACIFIC | PALMETTO | PALOMA | PATRIOT | PILGRIM | POLARIS |
|---|---|---|---|---|---|---|---|---|---|---|---|---|---|---|---|---|---|---|---|---|---|---|---|---|---|---|---|---|---|---|
| DRAPPER | 939 | | | | | | | | | | | | | | | | | | | | | | | | | | | | | |
| DUKE | 759 | 895 | | | | | | | | | | | | | | | | | | | | | | | | | | | | |
| EARLIBLUE | 763 | 805 | 609 | | | | | | | | | | | | | | | | | | | | | | | | | | | |
| ELISABETH | 831 | 1062 | 880 | 890 | | | | | | | | | | | | | | | | | | | | | | | | | | |
| ELLIOTT | 740 | 1040 | 817 | 865 | 830 | | | | | | | | | | | | | | | | | | | | | | | | | |
| GOLDTRAUBE71 | 867 | 1020 | 920 | 889 | 907 | 888 | | | | | | | | | | | | | | | | | | | | | | | | |
| HERBERT | 702 | 949 | 815 | 826 | 821 | 785 | 817 | | | | | | | | | | | | | | | | | | | | | | | |
| HORTBLUEPOPPINS | 905 | 1042 | 912 | 910 | 998 | 953 | 992 | 860 | | | | | | | | | | | | | | | | | | | | | | |
| IVANHOE | 764 | 898 | 747 | 600 | 892 | 854 | 917 | 776 | 957 | | | | | | | | | | | | | | | | | | | | | |
| JERSEY | 971 | 956 | 951 | 922 | 948 | 1033 | 1051 | 983 | 1041 | 1009 | | | | | | | | | | | | | | | | | | | | |
| JUBILIE | 1024 | 1066 | 1060 | 990 | 1109 | 1100 | 1106 | 1039 | 987 | 1060 | 1018 | | | | | | | | | | | | | | | | | | | |
| LATEBLUE | 634 | 962 | 751 | 785 | 810 | 753 | 855 | 481 | 844 | 787 | 997 | 986 | | | | | | | | | | | | | | | | | | |
| LEGACY | 951 | 1085 | 991 | 997 | 682 | 993 | 1009 | 904 | 914 | 996 | 861 | 1030 | 876 | | | | | | | | | | | | | | | | | |
| LIBERTY | 795 | 1058 | 902 | 936 | 896 | 620 | 928 | 838 | 983 | 905 | 1078 | 1103 | 815 | 1041 | | | | | | | | | | | | | | | | |
| MISTY | 971 | 1064 | 919 | 943 | 1077 | 1033 | 1083 | 985 | 1003 | 1027 | 956 | 1091 | 1017 | 1050 | 1084 | | | | | | | | | | | | | | | |
| MONDO | 853 | 999 | 932 | 929 | 998 | 962 | 1013 | 955 | 1066 | 947 | 1029 | 893 | 904 | 997 | 935 | 1035 | | | | | | | | | | | | | | |
| NEWHANOVER | 957 | 744 | 957 | 941 | 862 | 1034 | 1026 | 996 | 1036 | 984 | 933 | 1039 | 1022 | 871 | 1066 | 1032 | 1038 | | | | | | | | | | | | | |
| NORTHBLUE | 847 | 1085 | 961 | 934 | 1048 | 987 | 1025 | 978 | 1004 | 972 | 1084 | 1132 | 962 | 1125 | 1025 | 1168 | 1096 | 1113 | | | | | | | | | | | | |
| NORTHCOUNTRY | 993 | 1058 | 960 | 949 | 1058 | 1040 | 1036 | 1047 | 1062 | 999 | 1042 | 1092 | 1012 | 1107 | 1021 | 1156 | 1052 | 1095 | 942 | | | | | | | | | | | |
| NORTHLAND | 911 | 1068 | 890 | 916 | 979 | 936 | 942 | 843 | 951 | 866 | 1044 | 1115 | 878 | 1038 | 863 | 995 | 1041 | 1085 | 962 | 1008 | | | | | | | | | | |
| NUI | 965 | 1058 | 919 | 936 | 1080 | 1030 | 1068 | 986 | 994 | 1006 | 938 | 1070 | 1011 | 1035 | 1070 | 182 | 1008 | 1014 | 1165 | 1148 | 973 | | | | | | | | | |
| OZARKBLUE | 867 | 1116 | 983 | 1017 | 1013 | 994 | 994 | 955 | 1045 | 980 | 1015 | 1049 | 951 | 974 | 986 | 1084 | 967 | 1052 | 1031 | 1124 | 1034 | 1053 | | | | | | | | |
| O_NEAL | 941 | 115 | 899 | 804 | 1056 | 1031 | 1019 | 957 | 1042 | 896 | 962 | 1066 | 967 | 1085 | 1060 | 1059 | 1005 | 759 | 1097 | 1063 | 1076 | 1046 | 1117 | | | | | | | |
| PACIFIC | 841 | 1083 | 940 | 1020 | 955 | 906 | 854 | 847 | 1036 | 986 | 1068 | 1119 | 823 | 1051 | 928 | 1047 | 1032 | 1039 | 1064 | 1033 | 902 | 1035 | 986 | 1082 | | | | | | |
| PALMETTO | 902 | 1047 | 867 | 911 | 1048 | 986 | 1060 | 960 | 893 | 927 | 1022 | 989 | 954 | 946 | 999 | 963 | 961 | 1034 | 1110 | 1124 | 989 | 934 | 1010 | 1054 | 1051 | | | | | |
| PALOMA | 917 | 1001 | 967 | 966 | 1039 | 996 | 1054 | 955 | 1005 | 988 | 953 | 904 | 995 | 1068 | 1020 | 834 | 936 | 1025 | 1142 | 1143 | 1003 | 826 | 1071 | 1004 | 1039 | 956 | | | | |
| PATRIOT | 734 | 965 | 827 | 618 | 1005 | 924 | 996 | 879 | 872 | 788 | 1050 | 1134 | 867 | 1094 | 1020 | 1043 | 1043 | 1034 | 747 | 997 | 911 | 1041 | 1043 | 968 | 1051 | 999 | 1079 | | | |
| PILGRIM | 2419 | 2493 | 2477 | 2486 | 2447 | 2418 | 2447 | 2434 | 2478 | 2466 | 2464 | 2509 | 2436 | 2521 | 2410 | 2538 | 2479 | 2477 | 2469 | 2416 | 2459 | 2518 | 2519 | 2496 | 2430 | 2497 | 2503 | 2489 | | |
| POLARIS | 845 | 1086 | 949 | 938 | 1030 | 979 | 1067 | 995 | 1052 | 946 | 1095 | 1158 | 970 | 1089 | 1045 | 1132 | 1043 | 1107 | 746 | 960 | 1044 | 1142 | 1041 | 1096 | 1058 | 1103 | 1127 | 815 | 2459 | |
| REBEL | 982 | 897 | 993 | 987 | 1139 | 1087 | 1124 | 1031 | 1059 | 1033 | 969 | 1034 | 1062 | 1106 | 1104 | 961 | 951 | 985 | 1165 | 1087 | 1116 | 945 | 1089 | 902 | 1108 | 1020 | 927 | 1102 | 2493 | 1138 |
| REKA | 826 | 1001 | 867 | 801 | 915 | 924 | 931 | 837 | 988 | 853 | 1040 | 975 | 844 | 1012 | 973 | 1051 | 947 | 1019 | 943 | 944 | 954 | 1046 | 987 | 1009 | 976 | 1045 | 1034 | 939 | 2462 | 922 |
| ROXYBLUE | 870 | 675 | 871 | 814 | 933 | 956 | 968 | 878 | 999 | 892 | 998 | 1031 | 883 | 1030 | 998 | 1045 | 1001 | 889 | 1025 | 1025 | 1013 | 1042 | 1033 | 680 | 1024 | 1049 | 1034 | 995 | 2482 | 983 |
| RUBEL | 780 | 984 | 887 | 837 | 787 | 770 | 914 | 746 | 907 | 770 | 1016 | 1132 | 729 | 939 | 870 | 1099 | 980 | 1049 | 1034 | 1051 | 999 | 1103 | 987 | 983 | 1046 | 1026 | 1057 | 892 | 2460 | 1041 |
| SHARPBLUE | 983 | 1028 | 1067 | 956 | 1084 | 1032 | 1106 | 1062 | 1099 | 1045 | 993 | 729 | 1042 | 1064 | 1036 | 1036 | 683 | 1062 | 1184 | 1168 | 1138 | 1009 | 1113 | 1016 | 1144 | 1013 | 803 | 1100 | 2524 | 1166 |
| SPARTAN | 848 | 945 | 792 | 673 | 1008 | 960 | 950 | 915 | 995 | 858 | 1043 | 1076 | 880 | 1069 | 997 | 1064 | 979 | 1038 | 984 | 968 | 981 | 1058 | 1022 | 965 | 992 | 1012 | 1053 | 903 | 2496 | 1017 |
| STAR | 911 | 630 | 885 | 872 | 1069 | 1053 | 1052 | 982 | 1079 | 920 | 978 | 1088 | 1000 | 1082 | 1069 | 955 | 1020 | 934 | 1095 | 1035 | 1021 | 936 | 1082 | 642 | 1072 | 1038 | 972 | 1015 | 2437 | 1086 |
| SUNSHINEBLUE | 1085 | 1100 | 1108 | 1130 | 1188 | 1107 | 1137 | 1076 | 1112 | 1088 | 1067 | 1122 | 1117 | 1106 | 1101 | 896 | 1025 | 1121 | 1249 | 1199 | 1157 | 876 | 1139 | 1095 | 1110 | 1015 | 896 | 1207 | 2551 | 1254 |
| TOPSELF | 905 | 1022 | 786 | 884 | 1036 | 943 | 1034 | 985 | 1034 | 939 | 1005 | 1087 | 948 | 1051 | 1002 | 974 | 1006 | 1084 | 1039 | 1059 | 1003 | 963 | 1035 | 1020 | 976 | 1000 | 982 | 1008 | 2514 | 1087 |
| CENTRABLUE | 1474 | 1540 | 1537 | 1539 | 1505 | 1515 | 1509 | 1516 | 1539 | 1521 | 1482 | 1493 | 1512 | 1522 | 1532 | 1512 | 1525 | 1536 | 1537 | 1506 | 1543 | 1495 | 1473 | 1553 | 1504 | 1496 | 1514 | 1560 | 2583 | 1541 |
| COLUMBUS | 1480 | 1524 | 1522 | 1527 | 1496 | 1504 | 1497 | 1499 | 1539 | 1534 | 1497 | 1521 | 1504 | 1497 | 1521 | 1520 | 1532 | 1527 | 1509 | 1476 | 1538 | 1490 | 1457 | 1540 | 1510 | 1516 | 1549 | 1526 | 2597 | 1520 |
| OCHLOCKONEE | 1521 | 1562 | 1551 | 1562 | 1542 | 1517 | 1565 | 1538 | 1555 | 1541 | 1518 | 1533 | 1565 | 1539 | 1550 | 1539 | 1540 | 1547 | 1561 | 1503 | 1565 | 1522 | 1483 | 1567 | 1521 | 1515 | 1521 | 1556 | 2650 | 1561 |
| POWDERBLUE | 1546 | 1563 | 1592 | 1593 | 1615 | 1566 | 1595 | 1579 | 1604 | 1570 | 1544 | 1564 | 1598 | 1557 | 1598 | 1569 | 1549 | 1621 | 1609 | 1544 | 1609 | 1548 | 1510 | 1571 | 1575 | 1554 | 1570 | 1602 | 2679 | 1597 |
| SKYBLUE | 1507 | 1559 | 1561 | 1557 | 1541 | 1541 | 1532 | 1518 | 1550 | 1561 | 1516 | 1515 | 1542 | 1520 | 1588 | 1536 | 1528 | 1545 | 1558 | 1494 | 1587 | 1511 | 1483 | 1566 | 1526 | 1540 | 1542 | 1563 | 2618 | 1584 |
| TORO | 717 | 936 | 774 | 746 | 850 | 834 | 843 | 727 | 857 | 801 | 940 | 985 | 674 | 928 | 815 | 983 | 917 | 912 | 990 | 982 | 873 | 967 | 933 | 919 | 846 | 955 | 950 | 829 | 2399 | 984 |

| | REBEL | REKA | ROXYBLUE | RUBEL | SPARTAN | STAR | SUNSHINEBLUE | TOPSELF | CENTRABLUE | COLUMBUS | OCHLOCKONEE | POWDERBLUE | SKYBLUE | TORO |
|---|---|---|---|---|---|---|---|---|---|---|---|---|---|---|
| REKA | 1092 | | | | | | | | | | | | | |
| ROXYBLUE | 1014 | 641 | | | | | | | | | | | | |
| RUBEL | 1080 | 952 | 964 | | | | | | | | | | | |
| SHARPBLUE | 925 | 1099 | 1063 | 1079 | | | | | | | | | | |
| SPARTAN | 1036 | 929 | 921 | 968 | 1068 | | | | | | | | | |
| STAR | 923 | 1025 | 867 | 1019 | 1056 | 1019 | | | | | | | | |
| SUNSHINEBLUE | 977 | 1176 | 1180 | 1109 | 1012 | 1171 | 1069 | | | | | | | |
| TOPSELF | 1058 | 1017 | 1029 | 1039 | 1074 | 1025 | 1001 | 1045 | | | | | | |
| CENTRABLUE | 1499 | 1546 | 1556 | 1522 | 1510 | 1559 | 1526 | 1491 | 1552 | | | | | |
| COLUMBUS | 1518 | 1568 | 1564 | 1519 | 1515 | 1541 | 1511 | 1484 | 1527 | 1049 | | | | |
| OCHLOCKONEE | 1523 | 1579 | 1591 | 1543 | 1526 | 1564 | 1527 | 1485 | 1532 | 1172 | 1009 | | | |
| POWDERBLUE | 1559 | 1587 | 1596 | 1590 | 1537 | 1607 | 1534 | 1491 | 1588 | 1096 | 986 | 837 | | |
| SKYBLUE | 1516 | 1570 | 1582 | 1536 | 1532 | 1582 | 1536 | 1492 | 1584 | 769 | 1082 | 1138 | 1100 | |
| TORO | 1011 | 760 | 835 | 867 | 1078 | 829 | 964 | 1091 | 904 | 1530 | 1546 | 1540 | 1575 | 1539 |
