# Supplementary material for: Genetic diversity assessed by genotyping by sequencing (GBS) and for phenological traits in blueberry cultivars
Source: PLoS One. 2018 Oct 23;13(10):e0206361. doi: 10.1371/journal.pone.0206361 (PMC6198992; doi:10.1371/journal.pone.0206361)
Supplement: S4 Table — Tag sequence including the 29 most informative SNPs identified in the PCA analyses. The scaffold of the V. macrocarpon genome (available at www.vaccinium.org) which includes the tag sequence is indicated. The allele variation identified is indicated between brackets in red color (+, insertion; -, deletion). (PDF) [file pone.0206361.s005.pdf]

**Table S4.** Tag sequence including the 29 most informative SNPs identified in the PCA analyses. The scaffold of the *V. macrocarpon* genome (available at www.vaccinium.org) which includes the tag sequence is indicated. The allele variation identified is indicated between brackets in red color (+, insertion; -, deletion)

| SNP_name | Scaffold | tag sequence |
|---|---|---|
| S1_357262673 | 84476 | TGCATCTAA(T/G/+/-)TTATTGCTCCTCTAACCTGATGTACATCGTGATGCA |
| S1_111934477 | 10584 | TGCATGAC(C/A)AGTCACATAGGTAGGAACTTGCTTACAGTAATGAAAGTATTCATGCA |
| S1_200157800 | 25792 | TTTTAGCATTAAAACCACAATTA(T/C)GACTATCACCAAGCTGGATCAGGAGCACAAGCATTATGCA |
| S1_251730461 | 38656 | TGCATGTACTTTCAATAGACAATAC(G/A)ATGACAAATTTAACTCTCTTCCAGTATCCACGAATTAA |
| S1_271131719 | 44613 | TGCATGACCTGGGCTCC(T/C)CTGCTCCTTAGACTTTGGATATTGGACATTGCACTCCTATAAGTAT |
| S1_155082527 | 17155 | TCATAAAAATAGAAAT(C/T)CACATCACCTTCAATTTGCAAGCGAACAGAGACCTTAGGAATATGCA |
| S1_230369288 | 32864 | TGCATTTCAGCAGTTTTGGCAACAGTTTGAATCTGCAATGCG(A/G)CAGGTATGCA |
| S1_174636534 | 20657 | TGCATAGTTCATGG(C/T)GAATTTGAGCTGCAAAATATGCTTGCTAGTTGAATAGCTAACTTATCAG |
| S1_108191452 | 10083 | TGCATGG(G/T)TTCTCCTTGTTCAGTGCATCTTCAAAGAATTTATGGTTTTATTCTCTACCTCTTTT |
| S1_277080239 | 46596 | A(-/+)CACGCCCCCTGTTTACTATTTTATCCTTCCTTACTATTTTATCCATCCAAAACTATCATGCA |
| S1_393623070 | 116375 | TGCATA(C/T)GTGATTGTTGCAAGTTTTGATTTGATAGTTGTGTCGAAGTTTTGTTGCTGATGGCAA |
| S1_266222027 | 43034 | TGCATGGTAGTC(C/T)AGTGGTAGATACCATATGCTCCATGCA |
| S1_329095780 | 68072 | TGCATAT(G/C)TTACGCATGCAATATACGTAATTTGTATGCA |
| S1_300604427 | 55269 | TGCATACAAGGTAGCTTT(G/A)ATCTTCTTAAGGTCTGTTTGGGATAATAATTTTTCTGGGTTGTGG |
| S1_261116383 | 41448 | TGCATTTTCC(G/A)CCGTTGATGGATGTTTTGCCGAAAGACATGCA |
| S1_200638991 | 25895 | TGCATC(C/T/+/-)CAAAAAGTACATATCTCAAATTTAGTGCAGATTGCAGAACCTATAATGTTCAATCTC |
| S1_77725901 | 6372 | TGCATAGTGATTTGCGTGTA(T/+/-)GTGTTTTTGTACATTTGGATCAAGAACACCCATAGGATGCA |
| S1_180489707 | 21773 | (-/+)ACTTTTTTATAACTTAAATTTTGTTTGAACTGCATAAACATAAATTATCCCGTCACTCATGCA |
| S1_82331223 | 6893 | TGCATAA(T/C)TGACTTTACACCATCTTTAGTAACTTTTATGCA |
| S1_46187511 | 3191 | TGCATGGTAAACTTCACATAC(T/G)TGCAACCACACATGAGCCACAACTATCAGCTAAGTCAGAGTG |
| S1_5585216 | 234 | (C/-/+/A)CCTTAACATCCCAAGGTGAGGTTTTCCATCTCCTTTCAAAGTTTATTAGCCTGCTGTCATGCA |
| S1_295568253 | 53285 | TGCATC(A/G)GGACTCACCTCAGGACAAAGAACATTGACCCGAATTCCTTGTTTCTTGTATGGAACA |
| S1_26515316 | 1571 | TGCATTAGCA(A/T)ACATTACCGTGTTTGTTTGCATGCA |
| S1_279958037 | 47582 | CAATTTCCAGCACCGAACAAACAAATCATGTCGAGAGCAAACTTACTC(T/C)AGAATAGTATATGCA |
| S1_301146815 | 55487 | TGCATGT(A/T)ATTTTTGTGCTTATAAGTTCTAACTGGTTCCTTTTTTGCAAATGGATATGTAGAAA |
| S1_133635512 | 13704 | TGCATA(A/T)TTTTATCCAAATTAAACCACCACATTTGTCATTGTATATGCA |
| S1_210618315 | 28110 | TGCATCCA(C/T/+/-)TTACTAACACCTTCCGGTCCACATGCA |
| S1_29518046 | 1799 | TGCATAGTGAG(C/T)GCTCAAGCCTTGTTCATTCATGCA |
| S1_297197625 | 53918 | TGCATATT(C/T)TGCCCGATAACTGAGTCACAAACTAGTATGTCACCAGTGCCATGCACAGGTTTTG |
